# A Network Model of Resilience Factors for Adolescents with and without Exposure to Childhood Adversity

**DOI:** 10.1038/s41598-018-34130-2

**Published:** 2018-10-25

**Authors:** J. Fritz, E. I. Fried, I. M. Goodyer, P. O. Wilkinson, A.-L. van Harmelen

**Affiliations:** 1 Department of Psychiatry, University of Cambridge, Cambridge, England; 2 Department of Psychology, University of Amsterdam, Amsterdam, Netherlands; 3 Department of Clinical Psychology, Leiden University, Leiden, Netherlands

## Abstract

Resilience factors (RFs) help prevent mental health problems after childhood adversity (CA). RFs are known to be related, but it is currently unknown how their interrelations facilitate mental health. Here, we used network analysis to examine the interrelations between ten RFs in 14-year-old adolescents exposed (‘CA’; n = 638) and not exposed to CA (‘no-CA’; n = 501). We found that the degree to which RFs are assumed to enhance each other is higher in the no-CA compared to the CA group. Upon correction for general distress levels, the global RF connectivity also differed between the two groups. More specifically, in the no-CA network almost all RFs were positively interrelated and thus may enhance each other, whereas in the CA network some RFs were negatively interrelated and thus may hamper each other. Moreover, the CA group showed more direct connections between the RFs and current distress. Therefore, CA seems to influence how RFs relate to each other and to current distress, potentially leading to a dysfunctional RF system. Translational research could explore whether intervening on negative RF interrelations so that they turn positive and RFs can enhance each other, may alter ‘RF-mental distress’ relations, resulting in a lower risk for subsequent mental health problems.

## Introduction

Childhood adversity (CA) has been suggested to be “psychiatry’s greatest public health challenge” (p. e300)^1^. It is often assumed that adversities are unusual and uncommon experiences^2^, but large, population-representative research^3–5^ has shown that up to 53.4 percent of individuals under the age of 18 report having experienced at least one form of CA^5^. CAs span a wide range of severely stressful and traumatic experiences^3–5^, and account for more than a quarter of all mental health problems^3^. CAs can range from one-time events such as a loss of a significant other, a severe traffic accident, or sexual assault, to chronic experiences such as emotional neglect, physical maltreatment, or parental mental illness^6^. Given that CA poses a crucial risk to subsequent mental health problems, it is vital to examine how we can reveal and, where possible, facilitate mental health resilience in order to reduce the negative consequences of CA.

Mental health resilience describes the process of effective adaptation, i.e. staying mentally healthy, following adversity^6–10^. In other words, although CA increases the risk of mental illness, not all those exposed go on to develop mental health problems. Based on this concept, resilience factors (RFs) are defined as characteristics, skills and resources that reduce the risk of mental health problems subsequent to CA^6,11–13^. So far, resilience factors have most often been modelled as single main-, moderation-, and mediation-effects^6,9,14^. Resilience researchers have also started using growth curve models and predictive difference scores to aid the revealing and understanding of resilient functioning^2,9,15–17^. However, these approaches do not take into account that there are a range of RFs that are interrelated and potentially have combined effects, although it is commonly recognized that RFs do not function in isolation from each other^2,9,14,18–23^. For example, Boyes, Hasking and Martin^24^ showed that expressive suppression, cognitive reappraisal and rumination together mediate the association between a history of CA and mental distress. Crucially, no single RF has been reported as having a leading effect in benefitting mental health resilience^2,17^, which supports the conjecture that mental health resilience is better represented as an interrelated system of RFs.

Here, we aim to characterize the architecture of this system of RFs and its relationship with concurrent distress, in order to enhance our understanding of the putative mechanisms of RFs that may reduce the liability of poor mental health following CA. To this end, we apply network analysis, a statistical methodology that estimates and scrutinizes the unique interrelations among many variables at the same time (for a detailed, methodological rationale see Supplement I)^25^.

In the last few years, network analysis has been utilized as psychometric tool for the exploration of psychopathology^25–30^. In the present study, we will model the interrelations of selected RFs which are derived from our recent pre-registered systematic literature review^6^. To the best of our knowledge, this is the first time network analysis is used to estimate network models of RFs. Of note, we do not aim to study network-related ‘resilience’, e.g. the ability of a network to adjust flexibly to internal and external errors to remain functional^31^; rather, we aim to investigate how empirically-supported factors that enhance mental health resilience in adolescents following CA relate to each other. Thus, throughout the present article ‘resilience’ refers exclusively to ‘mental health resilience’.

We focus on RF networks in adolescence, a crucial developmental period for the first emergence of mental health problems^32^. First, we shall compare and contrast RF-RF interrelations between groups of adolescents exposed to (‘CA group’) and not exposed to CA (‘no-CA group’). Given that adolescents exposed to CA have on average a higher vulnerability to mental health problems than adolescents not exposed to CA^3^, we assume that this heightened vulnerability may go together with lower RF levels, and may influence how RFs interrelate. Second, we shall examine the influence of a general distress factor, indexing mental health problems, on the RF network models of the CA and the no-CA group. As the RFs included in the present study were empirically found to mitigate (i.e. moderate and/or mediate) the positive relationship between CA and mental health problems^6^, we expect that the general distress variable may differentially influence CA and no-CA group networks. With the suggested RF network analyses we aim to provide novel insights into the architecture of empirically supported RFs, and their relations with general distress, which may advance our understanding of the complex system of factors that improve mental health resilience.

## Aims and Hypotheses

We will study three consecutive research aims:

1. *Estimating RF network models*: We expect that RFs will be related to each other in both group networks, but that CA and no-CA networks will be dissimilar in structure.
2. *Estimating RF network models including a general distress index*: We will explore the impact of general distress levels on the network structures, through contrasting the CA and no-CA network structures after adding a general distress variable to the networks.
3. *Investigating potential group differences due to the influence of general distress on the network models:* We will further scrutinize whether potential differences in CA and no-CA networks, upon taking distress levels into account, result from (1) corrected ‘RF-RF’ interrelations, (2) ‘RF-general distress’ interrelations, or (3) both.

## Results

### Variable Preparation and Comparison between the CA and the no-CA Group

We recently carried out a preregistered systematic review of RFs^6^. Ten of the 20 identified RFs had been measured in the studied adolescent cohort (Roots^33^; *N* = 1238; girls = 674, boys = 564, age *M* = 14.49, *SD* = 0.28) and are included in the analyses. We estimated confirmatory factor analyses (CFAs) to compute factor scores for nine of the 10 RFs: High friendship support (i.e. part of social support), high family support, high family cohesion, high distress tolerance, high positive self-esteem, low negative self-esteem, low brooding, low reflective rumination, low aggression, and low expressive suppression. No CFA was performed for the single item expressive suppression RF. We used a general distress factor as index for concurrent mental health, which we derived from a previously published bifactor model obtained from self-reported scores for depression and anxiety symptoms^34^. Details of the CFAs, location and dispersion values, as well as further variable preparation details (e.g. transformation) can be found in Supplement II.

The CA group (*n* = 638, 56% girls) reported lower levels for nine of the 10 RFs when compared to the no-CA group (*n* = 501, 52% girls; see Table 1). Reflective rumination did not differ between the two groups. Furthermore, the CA group had higher general distress levels.

**Table 1:** RF and General Distress Comparisons: CA (n = 638) versus No-CA (n = 501) Groups.

Variable^*1/*2^	CA	no-CA	t^*3^/*X*^2*4^ (DF)	*p*	95% CI^*5^
Friendship support (high)	−0.07	0.06	2.23(1054.8)	0.03	0.02–0.25
Family support (high)	−0.08	0.09	2.79(1045.3)	0.01	0.05–0.29
Family cohesion (high)	−0.18	0.20	6.41(1066.4)	<0.001	0.27–0.50
Negative self-esteem (low)	−0.13	0.10	3.79(1071.5)	<0.001	0.11–0.35
Positive self-esteem (high)	−0.14	0.17	5.07(1070.9)	<0.001	0.19–0.42
Brooding (low)	−0.09	0.09	2.96(1046.4)	<0.005	0.06–0.30
Reflective rumination (low)	−0.06	0.01	1.21(1047.5)	0.23	−0.05–0.19
Distress tolerance (high)	−0.13	0.14	4.56(1072.4)	<0.001	0.16–0.39
Aggression (low)	low: 494 (score = 1) high: 119 (score = 0)	low: 435 (score = 1) high: 56 (score = 0)	12.51(1)	<0.001	
Expressive suppression (low)	low: 408 (score = 1) high: 209 (score = 0)	low: 366 (score = 1) high: 129 (score = 0)	7.56(1)	0.01	
General Distress	0.13	−0.16	−4.85(1049.4)	<0.001	−0.41–−0.17

*Note*. CA = childhood adversity. ^*1^All RFs are scored in such a way that high values are protective (e.g. high levels of high friendship support or high levels of low negative self-esteem) and low values are harmful (e.g. low levels of high friendship support or low levels of low negative self-esteem). ^*2^The continuous general distress variable is scored in such a way that the higher the value the higher the level of general distress. ^*3^We applied Welsh’s two-tailed independent sample t-test to account for potentially unequal variances across groups. ^*4^We applied two-tailed Pearson’s chi-square tests. ^*5^The confidence interval (CI) for the difference in location estimates, corresponding to the alternative hypothesis.

In the remainder of the article we report the results of the regularized partial correlation networks, which provide information about the unique interrelationships of two variables while correcting for all other variables^35–37^. Results of networks representing zero-order correlations can be found in Supplement III.

### Research Aim 1: RF Network Models

**Figure 1:**
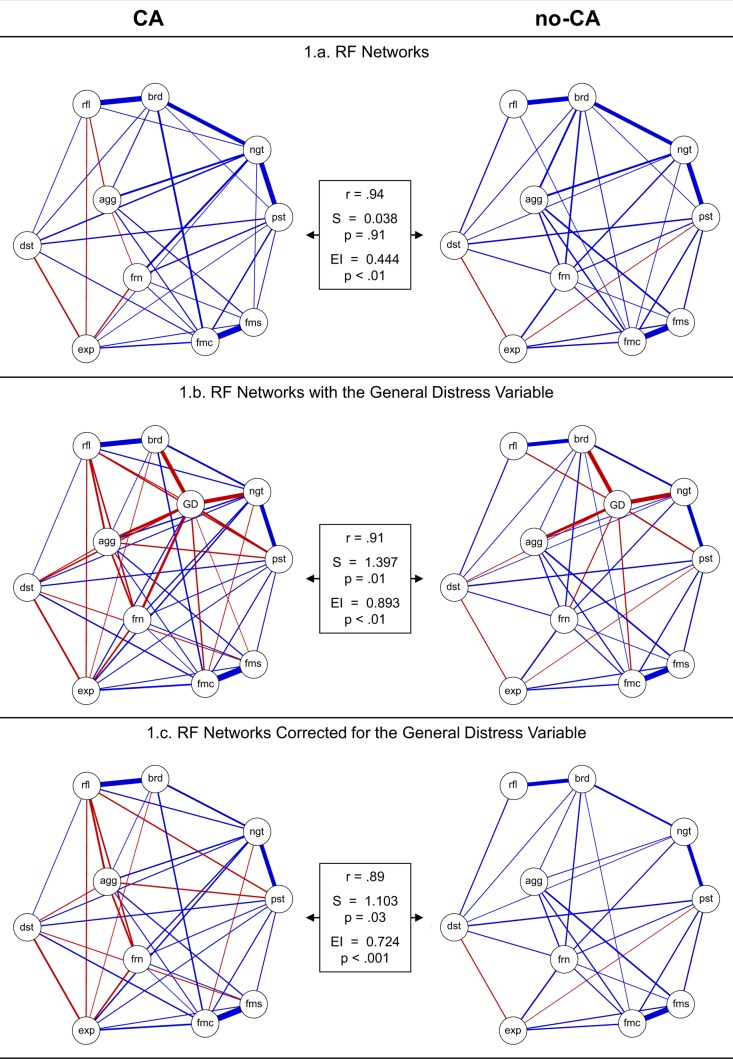
CA (n = 638) and no-CA (n = 501) resilience factor networks without (**a**) with (**b**) and corrected for (**c**) the general distress variable. Width of the lines = association strength. Positive interrelations = blue, negative interrelations = red. Legend: Frn = friend support, fms = family support, fmc = family cohesion, ngt = negative self-esteem, pst = positive self-esteem, rfl = reflection, brd = brooding, dst = distress tolerance, agg = aggression, exp = expressive suppression, GD = general distress. The boxes depict the adjacency matrix correlation between the respective two networks (r), the difference in global network strength between the respective two networks (S), the difference in global network expected influence (EI) between the respective two networks (EI), and the p-value corresponding to the global network strength and global network EI comparisons (5000 comparison samples). The above networks with faded interrelations can be found in Supplement VIII.

Firstly, we examined whether RFs are related to each other in both the CA and the no-CA group networks. Both networks (see Fig. 1a; or Supplement IV) indicated positive relationships between most RFs. Three of the 45 RF interrelations differed in sign between the two groups. For example, low expressive suppression was associated with *low* friendship support in the CA network, but with *high* friendship support in the no-CA network. A more detailed discussion of the interrelatedness of the RFs in the network models can be found in Supplement V. Robustness (see Supplement VI) and sensitivity analyses (see Supplement VII) indicated that the network models were stable and network parameters were estimated with a high accuracy.

Secondly, we investigated whether CA and no-CA RF networks are dissimilar in structure. Contrary to our hypothesis, the CA and no-CA group network structures were highly similar (i.e. correlation between the 45 regularized RF interrelations of each group; r = 0.94). Moreover, the network structure invariance test was not significant (*M* = 0.17, permutations = 5000, *p* = 0.21), and the CA and no-CA networks did not differ with regard to the global network strength (*S* = 0.038, *S*_CA_ = 3.528, *S*_no-CA_ = 3.566, permutations = 5000, *p* = 0.91). The global network strength is the absolute sum of all RF interrelations (i.e. treating all interrelations as positive) and indicates the overall RF network connectivity. However, the networks did differ with regard to the global network expected influence (EI; *EI* = 0.444, permutations = 5000, *p* < 0.01), which is the sum of all positive RF interrelations after subtracting the sum of all negative RF interrelations. Hence, the global network EI gives an indication of the degree to which RF are assumed to enhance each other, which was significantly higher in the no-CA compared to the CA group (*EI*_CA_ = 2.950, *EI*_no-CA_ = 3.394). We additionally compared all individual RF interrelations across the two networks, resulting in 45 Holm-Bonferroni corrected permutation tests: Only the RF interrelation between expressive suppression and friendship support differed significantly between the two networks (*E* = 0.17, permutations = 5000, corrected *p* < 0.01).

### Research Aim 2: RF Network Models Including a General Distress Index

To explore the impact of general distress levels on the CA and no-CA network structures, we next added the general distress variable to the networks (see Fig. 1b). In the no-CA group, all RFs were negatively related to general distress, except for expressive suppression and family support which were not related to general distress (Table 2a). In the CA group, all RFs were negatively related to general distress, except for expressive suppression which was positively related to general distress (shown in bold in Table 2a). Based on this unexpected finding for expressive suppression we performed further analyses (see Supplement IX) which showed that most results remained similar when removing expressive suppression from the network models.

**Table 2:** Relationships between the RFs and the General Distress Variable.

2.a. Regularized Partial Correlation Network										
CA	negative SE	brooding	aggression	positive SE	reflection	family cohesion	friend support	distress tolerance	family support	expressive suppression
no	−0.40	−0.37	−0.24	−0.10	−0.07	−0.07	−0.06	−0.02	0.00	0.00
yes	−0.38	−0.35	−0.23	−0.16	−0.05	−0.09	−0.20	−0.09	−0.01	**+ 0.06**
**2.b. Association Network (i.e. Zero-Order Correlations)**										
**CA**	**negative SE**	**brooding**	**positive SE**	**aggression**	**reflection**	**friend support**	**family cohesion**	**family support**	**distress tolerance**	**expressive suppression**
no	−0.74	−0.71	−0.52	−0.51	−0.46	−0.36	−0.36	−0.27	−0.21	−0.03
yes	−0.75	−0.71	−0.57	−0.36	−0.45	−0.37	−0.43	−0.33	−0.31	**+ 0.04**

*Note*. CA = Childhood adversity (yes: *n* = 638, no: *n* = 501). SE = Self-esteem.

When adding the general distress variable to the networks, the CA and no-CA network structures remained highly correlated (r = 0.91). Importantly however, the CA network structure invariance test was now significant (*M* = 0.20, permutations = 5000, *p* = 0.045), and networks also differed with regard to the global network strength (*S* = 1.397, permutations = 5000, *p* = 0.01), which was higher in the CA group (*S*_CA_ = 5.352, *S*_no-CA_ = 3.955). Along those lines, the global network EI was significantly lower in the CA compared to the no-CA group (*EI* = 0.893, permutations = 5000, *p* < 0.01; *EI*_CA_ = 0.307, *EI*_no-CA_ = 1.200). For single interrelation comparisons we found, in line with the networks without the general distress variable, that only the interrelation between expressive suppression and friendship support differed significantly between the two networks (*E* = 0.20, *N* permutations = 5000, corrected *p* < 0.001). Ergo, upon adding the general distress variable to the networks, CA and no-CA network structures differed not only with regard to the global network EI, but with regard to all examined structural measures. This finding may either be the result of (1) differing ‘RF-RF’ interrelations between the two groups when taking general distress into account, (2) differing ‘RF-general distress’ interrelations between the two groups, or (3) of both. Therefore, we further examined those options.

### Research Aim 3: Investigating Group Differences Due to the Influence of General Distress on the Network Models

#### Group differences due to the influence of general distress on ‘RF-RF’ interrelations

Firstly, we explored whether differing CA and no-CA network structures, after taking general distress into account, are the result of differing ‘RF-RF’ interrelations between the two groups. To this end we compared the RF network structures that are corrected for the variance of the general distress variable, but do not include ‘RF-general distress’ interrelations, between the CA and no-CA group. In other words, those networks contain only ‘RF-RF’ interrelations that are corrected for general distress levels, but do not contain the general distress variable itself (see Fig. 1c). The comparison of the resulting CA and no-CA network structures revealed a correlation of 0.89. Moreover, we found that the network structure invariance test was significant (*M* = 0.20, permutations = 5000, *p* = 0.04), that the networks differed with regard to the global network strength (*S* = 1.103, permutations = 5000, *p* = 0.03), which was higher in the CA group (*S*_CA_ = 3.744, *S*_no-CA_ = 2.641), and also with regard to the global network EI (*EI* = 0.724, permutations = 5000, *p* < 0.001), which was higher in the no-CA group (*EI*_CA_ = 1.790, *EI*_no-CA_ = 2.514). For single interrelation comparisons we again found a significant difference for the interrelation between expressive suppression and friendship support (*E* = 0.20, *N* permutations = 5000, corrected *p* < 0.01). Hence, RF-RF interrelations differ significantly between the CA and no-CA groups when correcting for general distress levels, both in terms of global network EI and global network strength (see Fig. 1c). Along those lines Fig. 1c. shows that in the no-CA network three RF-RF interrelations changed from positive to absent and all other interrelations kept the same relationship sign when being corrected for general distress levels. In contrast, in the CA network three RF-RF interrelations changed from positive to absent and seven interrelations changed from absent to negative. Moreover, the interrelatedness (or ‘centrality’) coefficients of the RFs also changed slightly in both the CA and the no-CA group, upon correcting for general distress levels (a discussion of those results can be found in Supplement X). Accordingly, differing CA and no-CA network structures, when taking general distress into account, are to some extent the result of general distress having a different impact on ‘RF-RF’ interrelations in the two groups.

#### Group differences regarding to ‘RF-general distress’ interrelations

**Figure 2:**
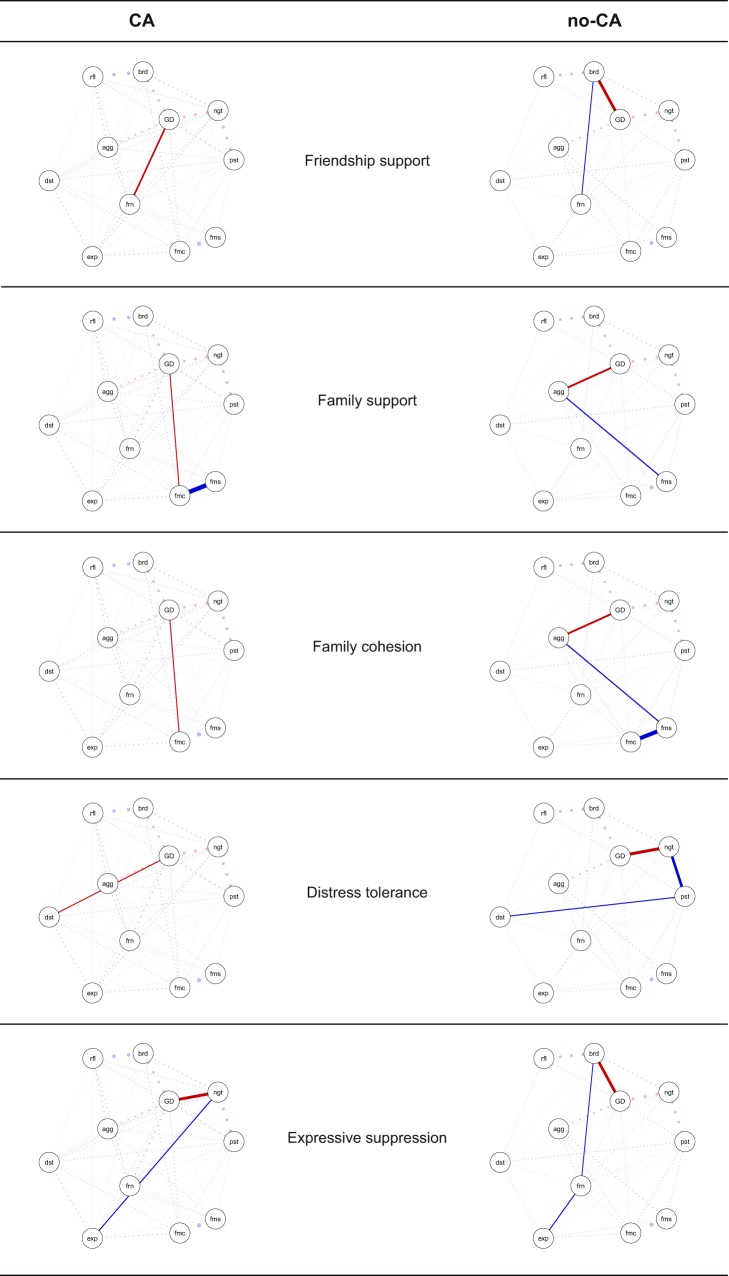
Shortest pathways between the resilience factors (RFs) and the general distress variable, that differed between the CA (n = 638) and the no-CA (n = 501) group. Non-transparent, continuous lines = shortest pathway of interest. Transparent, dotted lines = all remaining partial regularized correlation relationships. Positive interrelations = blue, negative interrelations = red. Legend: Frn = friend support, fms = family support, fmc = family cohesion, ngt = negative self-esteem, pst = positive self-esteem, rfl = reflection, brd = brooding, dst = distress tolerance, agg = aggression, exp = expressive suppression, GD = general distress.

To scrutinize whether differing CA and no-CA network structures, after taking general distress into account, are also the result of differing ‘RF- general distress’ interrelations between the two groups, we calculated the Shortest Path Lengths (‘shortest pathways’; see Fig. 2) between the RFs and general distress, and compared them between the groups. The shortest pathways indicate whether RFs have more direct or indirect connections with general distress (i.e. indirect connections go via intermediate RFs). Thus, a shortest pathway indicates the ‘quickest’ way to traverse the network from the RF to the general distress variable. In the CA group, six RFs had a direct shortest pathway with general distress, whereas in the no-CA group only three RFs had a direct shortest pathway. All other shortest pathways were indirect (for further details see Supplement XI). This finding was particularly interesting, as the regularized partial correlations between the RFs and general distress appeared to be rather similar in the two groups (Pearson r = 0.92; Spearman r = 0.88; or see Table 2a). Thus, the shortest pathways between RFs and general distress seemed for some RFs to differ between the two groups, despite the fact that the regularized partial correlations between the RFs and general distress were similar in the two groups. This may suggest that the differing ‘RF-RF’ interrelations of the two groups facilitate more direct and less indirect ‘RF-general distress’ pathways in the CA compared to the no-CA group. Accordingly, differing CA and no-CA network structures, after taking general distress into account, seem to result from differing ‘RF-RF’ interrelations, which in turn may lead to differing ‘RF-general distress’ pathways in the two groups.

## Discussion

CA has deleterious consequences on adolescent mental health, and understanding how RFs facilitate good mental health is a fundamental goal of resilience research. Here we estimated RF network models for groups of adolescents with and without CA, in order to establish the first “over-arching theoretical construction” (p. 605)^22^ of how RF systems may facilitate mental health after CA. We found that the degree to which RFs enhance rather than hamper each other (‘global network EI’) was significantly higher in the no-CA compared to the CA group. Upon the correction of distress levels, RF-RF interrelations of the two groups additionally differed with regard to the overall network connectivity (‘global network strength’). Moreover, interrelation pathways between RFs and concurrent general distress levels also seemed to differ between the two groups. Thus, differences between the CA and the no-CA groups seem to be underpinned by both differences in how RFs relate with each other, as well as by differences in how RFs relate to general distress.

When we only investigated RF interrelations, without taking general distress levels into account, the overall RF network connectivity (or ‘global network strength’) did not differ between adolescents with and without CA, and the maximal RF interrelation difference between the two groups (i.e. ‘network structure invariance test’) was also not significant. We revealed those findings despite that the mean levels of nine out of ten RFs were higher in the no-CA group than in the CA group. This may suggest that differing mean levels in RFs between groups do not necessarily lead to differences in the overall RF network connectivity between the groups. In a cross-sectional network model on depression symptoms, Schweren and colleagues^29^ did not detect a significant difference in the global network strength between strong- and weak treatment responders. Similarly, Snippe and colleagues^38^ applied dynamic network models on mental states prior to and after pharmacological as well as psychotherapeutic treatment for depression, and did not detect a global connectivity change in the pharmacological and only a marginal change in the psychotherapeutic treatment group, despite significant mean level reductions in depressive symptoms in both groups. Snippe and collegues^38^ tentatively concluded that the interrelations in the network models may represent an underlying ‘vulnerability’ to depression rather than relate to mean-level changes in symptoms and mental states. Translated to our findings, this may indicate that the interrelated RF system may represent some underlying form of the group-level ‘resilient functioning capacity’, regardless of mean-level differences in RFs between the two groups.

Interestingly, we found that in the CA network five RF interrelations had negative signs, which suggests that those RFs hamper rather than enhance each other. In the no-CA network, however, only two interrelations had a negative sign, of which one was also negative in the CA network. When we compared the two network structures regarding the global network EI, which indicates the degree to which RFs enhance rather than hamper each other, we accordingly found a higher level of enhancement in the no-CA compared to the CA group. This may be an indication for why adolescents exposed to CA have a higher liability of poor mental health^5^.

Our findings further suggest that after taking distress levels into account, interrelations of emotional, behavioural, cognitive and social RFs not only result in a higher degree of RF enhancement in the no-CA group, but also in a higher overall network connectivity in the CA group. More specifically, in the CA group, seven additional RF-RF interrelations were negative upon the correction of mental distress levels. Thus, whereas in the no-CA network almost all RF interrelations are positive and thus may enhance each other, in the CA network more than a quarter of the RF interrelations are negative and thus may hamper each other. Negative RF-RF interrelations in the CA network, upon the correction for distress levels, may further underpin a deficient functioning of the RFs, and thus may also be a reason for why adolescents with exposure to CA are on average more vulnerable for subsequent distress^6^.

Our findings additionally showed that the CA group had lower levels of RFs and higher levels of general distress. Therefore, a higher vulnerability to distress in the CA group may be substantiated by (1) high distress leading to lower RFs, (2) lower RFs leading to higher distress, or (3) by unfavourable, mutualistic ‘RF-mental distress’ associations (e.g. mutualistic coupling^39^). Moreover, the CA group had more direct connections between RFs and concurrent general distress, compared to the no-CA group. More specifically, in the no-CA group only three RFs had direct shortest pathways with general distress, whereas in the CA network six RFs had direct shortest pathways with general distress. In case of *high* RFs and *low* general distress, many direct ‘RF-distress’ pathways may be advantageous, as many high RFs then directly can contribute to lower distress levels (and/or vice versa). Yet, in case of *low* RFs and *high* distress, as in the CA group, many direct ‘RF-distress’ pathways may be disadvantageous, as high distress then directly can contribute to many low RFs (and/or vice versa). Hence, lower levels of RFs and higher levels of distress, together with more direct RF-distress relationships, may be another reason for why adolescents with exposure to CA are on average less protected from subsequent distress^6^.

Given our finding for the no-CA group, that almost all RFs were positively interrelated, it is likely that enhancing the most strongly connected RFs in the RF system may spread through the network and thereby enhance the level of other RFs. Many positively interrelated RFs that enhance each other, may in turn lower concurrent, and thus potentially also subsequent mental distress. In contrast, in the CA network many RF interrelations were negative. Therefore, enhancing RFs in the CA network may not be sufficient to effectively reduce distress levels, as higher levels of RFs may even further hamper other RFs. However, reducing general distress levels in the CA network could be achieved by intervening on negative RF-RF interrelations to turn them into positive interrelations, so that RFs enhance rather than hamper each other. Examining this should be subject of future research.

A potentially important negative RF-RF interrelation in the CA network is the ‘expressive suppression – friendship’ interrelation, which differed significantly from the corresponding positive RF-RF interrelation in the no-CA network. This finding suggests that, in the CA group, (1) ineffectively communicating emotions drives friendship withdrawal, (2) friendship withdrawal drives ineffectively communicating emotions, or (3) both drive each other reciprocally over time (reciprocal coupling). For example, it may be that CA exposure results in higher manifest levels of negative emotions^40^; and showing these emotions may burden friendships and/or reduce socializing behaviours in peers. Alternatively, an already existing low level of friendships and socializing may generate more negative emotions and thus support an increased expression of those emotions. Translational research could test whether training CA-exposed adolescents to communicate their own emotions better, may lead to improved friendships. If our finding of potentially dysfunctional RF interrelations in the CA group holds up in replication over time and in independent samples, this may explain why individuals with a history of CA are on average less likely to respond to treatment for mental health problems than individuals without a history of CA^41^.

Our study is not without practical limitations. First, CA was assessed retrospectively, which has the disadvantage of potential recall bias^33^. Second, CA was classified as a binary variable, categorizing ‘any’ versus ‘no’ history of CA. Such a categorization is rather crude, as it assumes that any form of adversity, irrespective of the severity and frequency, contributes to a difference in mental health between CA and no-CA groups. Instead, the effects of CA on the general distress variable may be linear (the more CA, the higher the probability of general distress) or U-shaped (e.g. challenge or inoculation theory; no or high CA goes together with high general distress, moderate CA goes together with low general distress)^9,14,42^. However, prior analysis of our adversity data demonstrated that, in our sample, CA could not be modelled as a single continuous variable, as a one factor CFA model did not fit the data^40^. Moreover, clustering CA in multiple classes would not have been possible in terms of power. Third, we mainly measured family-related adversities, which may limit the generalization to other types of CAs such as peer to peer bullying. Fourth, only a subset of empirically supported RFs^6^ was measured in Roots. The restricted number of included RFs may impact the network structure and may limit the content validity. Fifth, all RFs were solely assessed after the exposure to and the assessment of CA. Therefore, the study design does not allow for the establishment of baseline RF interrelations prior to CA. Ergo, we cannot draw conclusion with regard to the extent to which RFs change from pre to post CA^17,43^. Sixth, some variables had missing data. This led in the complete-information samples to 11.58 percent less data for the no-CA group and 20.38 percent less data for the CA group, when compared to the respective full-information samples for CA and no-CA groups. Seventh, our data modelling procedure was conducted in two steps: (a) deriving RF scores from polychoric CFAs and (b) estimating network models for the resulting RFs. Future studies may look into latent network modelling, which is a novel methodology that efficiently performs both steps at one time^44^.

Our study also contained theoretical limitations. First, as all estimated networks were cross-sectional, the general distress variable was assessed at the same time as the RFs. Hence, it is likely that the psychological state of the adolescents influenced their self-ratings (and parent-ratings) of RFs. Therefore, the network models with the general distress variable mainly serve as a proof of principle, to check that the RFs are indeed related to general distress. It is important that future studies investigate the predictive values of the RFs, through scrutinizing the interrelations between RFs and subsequent general distress. Second, as our expressive suppression factor was assessed with only one item, our expressive suppression factor may have lacked specific aspects of the concept (i.e. content validity), or we may have measured a different construct than prior research (i.e. construct validity). Tapping a potentially different aspect of expressive suppression may explain our contrasting findings with the literature^24,45^ (i.e. we found that in the CA group the expressive suppression RF had a positive relationship with the general distress variable) and requires clarification in future studies. Yet, removing expressive suppression from the network models did only slightly alter our findings. Third, it is interesting to note that, in the CA network, some RF-RF interrelations are negative upon controlling for the general distress variable. The explanation we put forward for this finding is that the result is due to different network structures in the CA and no-CA groups. Alternative statistical explanations for this result exist, such as conditioning on a collider. Conditioning on a collider (in this case general distress) can induce spurious negative relationships among variables^46^, similar to what we observed in the CA network once entering general distress. However, given that this only occurred in the CA network, despite rather similar ‘RF-general distress’ (regularized partial) correlations in the two groups, conditioning on a collider does not plausibly seem to be the main explanation for the negative RF interrelations, as one would expect this to happen in an equal manner in the no-CA network (for further discussion see Supplement XII). Yet, in our sample eight of the 10 RFs functioned as mediators (indirect effects) and one additionally as moderator (interaction effect) for the relationship between CA and general distress, which may perhaps help explain why the correction for distress levels had differing effects on the RFs in the CA compared to the no-CA group (see Supplement XII). Fourth, it is crucial to note that our findings are derived from group level analyses, and thus represent averages across all participants. Therefore, our findings may not directly translate to person specific levels and thus may not apply to all adolescents with CA. For clinical purposes, RF interrelations should be evaluated on an individual level.

Besides those limitations, our study also has notable strengths. For example, our study combines several advanced statistical methods - i.e. categorical CFAs, latent class analysis, bifactor modelling, and network analysis - and thereby accomplished to be the first study to model a complex system of RFs. Moreover, as all included RFs were empirically found to moderate and/or mediate the positive relationship between CA and mental health problems^6^, we believe that our RF models represent the construct we intended to measure well and thus achieved high construct validity.

To the best of our knowledge, this is the first time that network analysis has been applied to establish the interrelatedness of empirically supported RFs. We draw several conclusions aimed at aiding the refinement of resilience theory as well as the development of translational research regarding mental health resilience following CA. Yet, our findings require replication across time and in independent samples. Our findings suggest that the degree to which RFs enhance rather than hamper each other (‘global network EI’) was significantly higher in the no-CA compared to the CA group. Moreover, upon correction for general distress levels, the RF networks additionally differed with regard to the global RF connectivity. More specifically, in the no-CA network almost all RFs were positively interrelated and thus may enhance each other, whereas in the CA network some RFs were negatively interrelated and thus may hamper each other. Moreover, the CA group showed more direct relations between RFs and the general distress variable. Thus, differences between the CA and the no-CA groups seem to be underpinned by both differences in how RFs relate with each other, as well as by differences in pathways between RFs and general distress. Translational research could explore whether intervening on negative RF-RF interrelation, so that they turn positive and RFs can enhance each other, may alter ‘RF-mental distress’ relations, resulting in a lower risk for subsequent mental health problems.

## Methods

### Design

Roots is a large-scale adolescent cohort (total *N* = 1238) in which 14-year-olds from 18 schools in Cambridgeshire were assessed (UK; 2005 to 2006). Before participation the adolescents and their caregiver had to provide written informed consent. The aim of the Roots study was to measure risk and resilience factors, in an attempt to predict and understand the development of psychopathology^33^. The study was confirmed by the Cambridgeshire Research Ethics Committee (No: 03/302) and was conducted in line with the Declaration of Helsinki as well as Good Clinical Practice guidelines.

### Sample

We included all adolescents who had complete data for CA (total *N* = 1139; CA *n* = 638; no-CA *n* = 501). The sample included 620 girls and 519 boys. The adolescents had a mean age of 14.49 years (*SD* = 0.28, range: 13.88–15.28). Neither gender nor age differed between the CA and the no-CA group (see Table 3). Adolescents in the CA group had more often a psychiatric history, and on average a lower SES, and higher levels of depression and anxiety symptoms than adolescents in the no-CA group (see Table 3).

**Table 3:** Sample Comparisons: CA (n = 638) versus No-CA (n = 501) Groups.

Variable	CA	No-CA	t^*1^/z^*2^/*X*^2*3^ (DF)	*p*	95% CI^*4^
gender	n girls = 358 n boys = 280	n girls = 262 n boys = 239	1.50(1)	0.22	
age	M = 14.49, SD = 0.28	M = 14.48, SD = 0.28	−0.43(1049.3)	0.67	−0.04–0.03
SES^*5^	n hard pressed = 77 n moderate means = 36 n comfortably off = 170 n urban prosperity = 37 n wealthy achievers = 318	n hard pressed = 30 n moderate means = 11 n comfortably off = 105 n urban prosperity = 41 n wealthy achievers = 314	5.45	<0.001	
psychiatric history (PH)^*6^	n PH = 201 n no-PH = 437	n PH = 74 n no-PH = 427	42(1)	<0.001	
depression symptoms^*7^	M = 17.42, SD = 11.61	M = 14.03, SD = 10.46	−5.10(1088.5)	<0.001	−4.69–−2.09
anxiety symptoms^*8^	M = 16.92, SD = 12.61	M = 13.92, SD = 11.28	−4.17(1089.2)	<0.001	−4.42–−1.59

*Note*. CA = childhood adversity. SES = socio-economic status. ^*1^We applied Welsh’s two-tailed independent sample t-test to account for potentially unequal variances across groups. ^*2^As SES was split in five ordered categories, we applied the two-tailed Asymptotic Cochran-Armitage test^70^. ^*3^We applied two-tailed Pearson’s chi-square tests. ^*4^The confidence interval (CI) for the difference in location estimates, corresponding to the alternative hypothesis. ^*5^SES was assessed with the ACORN classification system (http://www.caci.co.uk)^71^. ^*6^Psychiatric history was assessed with the Schedule for Affective Disorders and Schizophrenia for School-Age Children (Present and Lifetime Version), additionally including learning disabilities, clinical sub-threshold diagnoses and deliberate self-harm^72^. ^*7^Depression symptoms were assessed with the Mood and Feeling Questionnaire^47^. ^*8^Anxiety symptoms were assessed with the Revised Children’s Manifest Anxiety Scale^48^.

### Measures

Detailed information on reliability and validity of all measures can be found in Supplement XIII.

#### Childhood adversity (CA)

CA was assessed at age 14, with the semi-structured Cambridge Early Experiences Interview (CAMEEI)^40^ being conducted with the adolescent’s main caregiver (96% maternal report). The following topics were assessed: Family loss, family discord, atypical parenting style, lack of maternal affection/engagement, periods of unemployment, financial difficulties, parental/sibling psychiatric illness, parental/sibling medical illness with impact, sexual/emotional/physical abuse, criminality amongst family members, acute social disturbances, and chronic social difficulties^40^. The interview focussed on three timeframes (early childhood (EC): 0 to 5 years; later childhood (LC): 5 to 11 years; early adolescence (EA): 11 to 14 years) with the aim to enhance recall quality and to reduce the risk of recall bias^40^. In a previous report on this sample, Dunn and colleagues^40^ clustered adolescents based on their CA experiences using latent class analysis (LCA). They revealed four CA classes: Low CA (EC = 68.8%, LC = 59.3%, EA = 64.4%), moderate CA (EC = 18.7%, LC = 25.5%, EA = 21.7%), severe CA (EC = 5.8%, LC = 10.0%, EA = 6.9%), and atypical parenting CA (EC = 6.7%, LC = 5.2%, EA = 7.0%). The four latent classes revealed good class assignment accuracies, ranging from 79 to 95 percent, and the risk of psychopathological distress increased with the adversity intensity of the classes, indicating discriminant validity of the classes^40^. To ensure sufficient analytic power and consistency with previous reports on this sample, we split the adolescents in two CA groups: Group 1 in which the adolescents belonged to the low CA class for all time intervals (i.e. no-CA group, 44%), and group 2 in which the adolescents belonged to a class other than low CA for at least one time interval (i.e. CA group, 56%).

#### General distress

Depression symptoms were assessed with the Mood and Feelings Questionnaire (33 items)^47^. Anxiety symptoms were assessed with the Revised Children’s Manifest Anxiety Scale (28 items)^48^. In a previous report on this sample, a bifactor model of these sixty-one items revealed one latent factor termed the general distress factor and three specific group factors (hopelessness/suicidal thoughts, generalized worrying, and restlessness/fatigue)^34^. Here we exclusively utilize the general distress factor, as this single measure revealed the highest measurement precision (i.e. lowest conditional standard error of measurement), and all items except two loaded well on it^34^. A further report showed that severe mental illness symptoms also loaded well onto the general distress factor^49^. Moreover, the general distress factor has good external validity, being replicated in two additional large-scale cohorts^49,50^.

#### Resilience factors (RFs)

Roots included the following RFs, all assessed via adolescent self-report (unless stated otherwise):

##### High friendship support

We used five items of the Cambridge Friendships Questionnaire^51^ to assess friendship support (e.g. ‘Can you confide in your friends?’)^51^.

##### High family support and high family cohesion/climate

We used five items of the McMaster Family Assessment Device^52^ to assess family support (e.g. ‘In times of crisis we can turn to each other for support.’), and the remaining seven items to assess family cohesion/climate (e.g. reversed: ‘We don’t get along well together.’)^52^. To support readability we will refer to cohesion when meaning cohesion/climate.

##### High positive and low negative self-esteem

We used the Rosenberg self-esteem scale^53^ (10 items) to assess positive self-esteem (5 items; e.g. ‘I was satisfied with myself.’) and negative self-esteem (5 items; e.g. ‘I felt that I was a failure.’)^53^.

##### Low reflective rumination and low ruminative brooding

We used five items of the Ruminative Response Scale^54^ (RSS) to assess reflective rumination (e.g. ‘I go away by myself and think about why I feel this way’)^54^. We used five items of the RRS^54^ (e.g. ‘I think about a recent situation, wishing it had gone better.’) and two items of the Short Leyton Obsessional Inventory^55^ (LOI; e.g. ‘I kept thinking about things that I had done because I wasn’t sure whether they were the right things to do.’) to assess brooding^54,55^.

##### High distress tolerance

We used five items of the Emotionality Activity Sociability Temperament Survey^56^ to assess distress tolerance (e.g. reversed: ‘He/she reacts intensely when upset.’; note: parent report)^56^.

##### Low aggression

We used four items of the Behaviour Checklist^57^ (11 questions based on the DSM-IV^58^ criteria for conduct problems) to assess aggression (e.g. ‘I have deliberately hurt or been cruel to an animal (e.g. a pet).’)^57^.

##### Low expressive suppression

We used one item of the Antisocial Process Screening Device^59^ to assess expressive suppression (i.e. ‘Does not show feelings or emotions.’; note: parent report)^59^.

### Analysis

#### Variable preparation

Firstly, we computed the ten above described RFs. Nine of the ten RFs were computed with one-factor confirmatory factor analyses (CFAs). As the items were assessed on an ordinal measurement level, we estimated the CFAs based on polychoric correlations, using the lavaan package in R^60^. All CFAs provided an acceptable fit to the respective items (for details see Supplement II). For expressive suppression, we used a standardized item score, as this RF was assessed with a single item. Secondly, we prepared the RFs for the network analysis. To reduce deviations from normality we applied the nonparanormal transformation to the RFs and the general distress variable (R package: huge)^61^. To meet the exchangeability assumption of permutation tests, which we used to compare the CA and the no-CA group networks (those tests are explained in depth below), we transformed variables for the overall sample before splitting the sample into CA and no-CA adolescents, to ensure that an RF has the same scale in the CA and the no-CA group. Moreover, we dichotomized variables that had a substantially restricted range (i.e. expressive suppression and aggression RFs).

#### Network estimation

We estimated the network models separately for the CA and the no-CA groups. In the visualization of the network models, the RFs are depicted as circles, called ‘nodes’ (or ‘vertices’; see Fig. 1). Nodes are connected by lines, called ‘edges’ (or ‘links’). The thickness of the edges indicates to what degree RFs are related, and the color of the edges indicates the relationship sign (i.e. positive = blue, negative = red)^35,62^. Cross-sectional networks can for example represent zero-order correlations (association network) or regularized partial correlations between RFs. The regularized partial correlation network provides information about variable interrelations after controlling for all other included variables.

Those network models estimate many interrelations, leading to the risk of false positive interrelations^35–37^. To prevent this, we used the least absolute shrinkage and selection operator (LASSO) regularization method. The LASSO sets weak partial correlations to exactly zero, almost always resulting in a sparse network^35,36,62,63^. We applied LASSO regularization rather than significance values, as significance levels have an arbitrary threshold as well as either the disadvantage of multiple testing problems or lower power when applying multiple testing corrections (for further explanation see^35^). To obtain the interrelations between variables, we used the cor_auto function^28^ in R that estimates the appropriate correlation type: Pearson for two continuous variables, polychoric for two dichotomous variables, and polyserial for one continuous and one dichotomous variable.

#### Network inference

Based on the estimated RF network, interrelatedness (or ‘centrality’) coefficients can be calculated, which help to interpret the results of the network model. We calculated three coefficients. *Node strength* is the sum of the interrelation values (e.g. regularized partial correlations) of a given RF with all directly related RFs (i.e. the sum of the *absolute* values of the RF interrelations)^35,62^. *Expected influence* is based on the formula of node strength, but takes negative relationships between RFs into account (i.e. the sum of the *relative* values of the RF interrelations)^62^. *Node predictability* is defined as the amount of variance of each RF that is explained by the directly related RFs. Node predictability is an *absolute* metric ranging from zero to 100 percent explained variance. Note, for dichotomous RFs, we based the node predictability on the normalized accuracy, instead of on the variance explained^64^. For a detailed discussion of these results see Supplement V and X.

#### Network stability and accuracy

To scrutinize the robustness of the estimated network models, we examined their stability and accuracy. Accuracy can be scrutinized through calculating nonparametric bootstrap confidence intervals (CIs, 95%) for the RF interrelations. The widths of these CIs give an indication for accuracy. Stability can be scrutinized through re-calculating interrelatedness coefficients such as the node strength for sample subsets. If the node strength remains similar in the subsets, this indicates that the RF network is stable^65^. Accordingly, we bootstrapped the RF interrelations (i.e. accuracy) and applied a subset bootstrap on node strength and expected influence (i.e. stability), with 2000 bootstraps each. For a detailed discussion of these results see Supplement VI.

#### Sensitivity analyses

To allow for the largest possible sample size, we based the network models on the *full-information* sample (*N* CA = 638; *N* no-CA = 501), using complete pairwise cases. As sensitivity analysis, we correlated the RF interrelations of the *full-information* networks with the RF interrelations of the *complete-information* networks (*N* CA = 508; *N* no-CA = 443), which are based on listwise case deletion (which was applied in previous research, see for example^66^). A high correlation would indicate that results are similar for both methods, and thus would support the soundness of *full-information* networks. For a detailed discussion of these results see Supplement VII.

#### Comparing CA and no-CA networks

To investigate the similarity of the CA and no-CA network structures, we calculated the correlation of the RF interrelations of the two groups (i.e. CA and no-CA network structure correlation). To examine the differences of the CA and no-CA network structures, we applied four permutation tests (i.e. two-tailed)^67^: Firstly, we tested whether the largest RF interrelation difference of the two networks (i.e. maximal edge weight difference) differs from the largest RF interrelation differences of randomly permuted network pairs, which functions as a network structure invariance test. Secondly, we tested whether the global network strength, i.e. the absolute sum of all RF interrelations, differs between the two network models (i.e. compared to permuted network model pairs). The global network strength indicates the overall network RF connectivity. Thirdly, we tested whether the global network expected influence (EI), i.e. the sum of all positive RF interrelations after subtracting the sum of the negative RF interrelations, differs between the two network models (i.e. compared to permuted network model pairs). The global network EI indicates the degree to which RFs enhance rather than hamper each other. Fourthly, we tested whether individual RF interrelations differ between the two networks (i.e. compared to the same individual RF interrelation differences between permuted network model pairs; please note, results for those tests without Holm-Bonferroni correction can be found in Supplement XIV)^67^.

#### Influence of the general distress variable on the RF networks

To investigate the relationship between the RFs and an index that underpins mental health problems, we added the general distress variable to the networks. We then compared the resulting networks between the CA and no-CA groups, by correlating the network structures between the two groups and by using the above described permutation tests. Moreover, we examined whether potential differences between the CA and the no-CA networks, upon taking the general distress variable into account, result from (1) differences in ‘RF-RF’ interrelations, (2) differences in ‘RF-general distress’ interrelations, or (3) from both. To test whether group differences may result from changes in ‘RF-RF’ interrelations, we tested whether ‘RF-RF’ interrelations that are corrected for general distress levels (i.e. networks corrected for distress levels, but this time excluding ‘RF-general distress’ interrelations), differ significantly between the two groups. This comparison was again conducted through correlating the network structures of the two groups and through using the above described permutation tests. To test whether group differences may result from differences in ‘RF-general distress’ interrelations, we computed the Shortest Path Lengths (‘shortest pathways’) between the RFs and the general distress variable (i.e. the inverse of the absolute interrelation(s) between the respective RF and the general distress variable)^35,66^. The shortest pathway between two variables indicates the direct or indirect connection between those two variables along the strongest connection(s), or in other words the ‘quickest’ way to traverse the network from the one variable to the other. Therefore, shortest pathways help to examine which RFs are mainly directly related to the general distress variable and which indirectly via other RFs. All network analyses were performed with the packages qgraph^28^, mgm^68^, bootnet^65^ and ‘NetworkComparisonTest’ (NCT)^67^, using R version 3.5.0^69^ in RStudio version 1.1.453.

### Code Availability Statement

Code supporting the findings of this study is available from http://jessica-fritz.com/.

## Electronic supplementary material

Supplement File

## Data Availability

Data for this specific paper has been uploaded to the Cambridge Data Repository https://doi.org/10.17863/CAM.20806 and is password protected. Our participants did not give informed consent for their measures to be made publicly available, and it is possible that they could be identified from this data set. Access to the data supporting the analyses presented in this paper will be made available to researchers with a reasonable request to openNSPN@medschl.cam.ac.uk.
