# Supplementary material for: A Network Model of Resilience Factors for Adolescents with and without Exposure to Childhood Adversity
Source: Sci Rep. 2018 Oct 25;8:15774. doi: 10.1038/s41598-018-34130-2 (PMC6202387; doi:10.1038/s41598-018-34130-2)
Supplement: Supplementary file 1 — Supplement File [file 41598_2018_34130_MOESM1_ESM.pdf]

Supplements for the Article:

A Network Model of Resilience Factors for Adolescents with and without Exposure to Childhood Adversity

J. Fritz[1,*], Dr. E. I. Fried[2,3], Prof. I. M. Goodyer[1], Dr. P. O. Wilkinson[1,A] & Dr. A.-L. van Harmelen[1,A]

* Correspondence: Jessica Fritz, jf585@cam.ac.uk

[1] University of Cambridge, Department of Psychiatry

[2] University of Amsterdam, Department of Psychology

[3] Leiden University, Department of Clinical Psychology

[A] shared last authorship

Supplement I

Methodological Rationale

The network models we estimated for the current article are based on (a) correlations between the RFs (see Supplement III) and (b) regularized partial correlations between RFs (see Figure 1 in the article). The article focuses on the discussion of regularized partial correlation networks for two reasons. First, partial correlations between RFs indicate to which extent two RFs are associated with each other, while controlling for all other RFs in the network. This way, we get an indication about which RFs predict each other and to which extent.[1] Second, we applied regularization to the partial correlations, as no partial correlation between RFs will be exactly zero.[1] Thus, regularization was applied to set very small partial correlations, which are likely to be false positives, to exactly zero (i.e. those interrelations are not depicted in the networks), resulting in potentially sparse models that exclusively depict the meaningful RF interrelations.[1]

To obtain the partial correlations for the discussed networks, it is necessary to (1) estimate the variance-covariance matrix, (2) take the inverse of this matrix (called precision matrix), and (3) standardize the precision matrix.[1] The standardized precision matrix then contains the partial correlations between the RFs, corrected for the respective other RFs in the matrix. In statistical terms, the corresponding graphical model for normally distributed, continuous variables is the Gaussian Graphical Model (GGM). Epskamp and colleagues[1] have shown that the GGM network model is closely related to least-squares regression. Similarly, GGM and structural equation models (SEM) are quite comparable as they both entail a constrained covariance structure.[2] Moreover, path models resemble network models with directed associations between variables (i.e. directed edges).[2] Furthermore, Epskamp and colleagues[3] have shown that network models based on dichotomous data, estimated as Ising models, can reveal probability distributions that are statistically equivalent to the distribution of latent variable models (i.e. multidimensional Item Response Theory (MIRT) models).

Accordingly, in specific situations, network models can be equivalent to latent variable models and comparing methodologies of network models and (at the least) similar statistical models is thus not sufficient to justify our methodological choice. Yet, we believe that network models have several practical, as well as theoretical advantages for our research aim ('to estimate and compare the interrelated system of RFs for groups of adolescents with and without a history of adversity'). First of all, network models (as opposed to other methods such as SEM) not only can estimate many variable interrelations (or in our case factor score interrelations) at the same time, but can also visualize those in form of a network graph. In our opinion, those graphs are highly insightful, as they enable the reader to process the interrelation strength and connectivity patterns of many RFs at the same time. Second, network analysis also enables the calculation of coefficients that indicate which variables are most central (e.g. most strongly interrelated with other variables) in the model, which can be seen as straightforward summary metrics that supplement the network graphs. Notably, those interrelatedness (or 'centrality') coefficients are usually not established and/or facilitated with SEM or path models. Third, and potentially most importantly, we believe that the underlying theory that nurtures our models – namely that RFs, which are empirically found to help prevent psychopathological distress after adversity, may not necessarily function in isolation, but may function as a complex interrelated system – goes along well with the theory of network modelling. Generally, network modelling puts the focus on the studied variables (or nodes) themselves,[4] in our case the RFs, whereas for example latent variable models put the focus on an underlying latent concept that explains or is explained by the studied variables,[4] e.g. such as an overall score for resilient functioning. Therefore, we think that network modelling facilitates the analysis of our specific research aim ('to estimate the interrelated system of RFs for groups of adolescents with and without a history of adversity (i.e. '*exploratory*') and to compare the two group networks with each other (i.e. '*confirmatory*')), particularly well. Last, we would like to highlight that our manuscript includes several methodological techniques in addition to network modelling, which are all chosen based on the specific analysis goal. I.e.

our CA variable is derived from a latent class analysis, our RFs are estimated with confirmatory factor analyses, and our general distress variable is estimated with a bifactor model.

Supplement II

Variable Preparation

The results of the polychoric confirmatory factor analyses (CFAs) for the RFs can be found in Table 1. We used the resulting latent factor scores of the RFs (i.e. standardized scores) as variables in the RF networks. We included recommendations from modification indices only if the suggestion could be theoretically underpinned, i.e. only if the suggested covariance was based on two similar worded items. Moreover, when items or item covariances led to negative (residual) variances, the respective item/covariance was removed from the CFA. This was done, as for models with negative (residual) variances factor scores cannot be established. For expressive suppression we used a scaled item score as variable (*n* = 1146), because expressive suppression was based on a single item.

The one-factor CFA for self-esteem[5] revealed a poor fit, even after the addition of two item covariances (Robust CFI = 0.96, Robust TLI = 0.94, Robust SRMR = 0.07, Robust RMSEA = 0.15, RMSEA 90% CI = 0.14 – 0.15). Based on prior research we established a two factor CFA model, resulting in a positive and a negative self-esteem factor.[6] Importantly, in a multiple-factor CFA we could not allow for covariances between factors. Allowing covariances between factors leads to inter-dependent factor scores. However, variables in networks cannot be based on inter-dependent scores, given that the aim of network analysis is to scrutinize the interrelation of variables and scrutinizing the interrelation of inter-dependent variables would be double dipping. Therefore, we established two one-factor models for positive and negative self-esteem, albeit being aware that the two models measure topologically similar concepts.[6]

Based on Treynor, Gonzalez, and Nolen-Hoeksema's[7] findings, we excluded 12 of the 22 RRS (i.e. rumination) items that overlapped with validated depression items (i.e. items of the Beck Depression Inventory[8,9]) and utilized two separate rumination factors. Respectively, one rumination factor for brooding[7,10] and one for reflection[7]. For the same reason as for self-esteem, we established two one-factor CFAs for rumination.

Table 1

Polychoric Confirmatory Factor Analyses Conducted with the WLSMV Estimator

**Robust CFI**	**Robust TLI**	**Robust SRMR**	**Robust RMSEA**	**RMSEA 90% CI**
Friendship support[11], *1 factor, 5 items, 1 additional item covariance, n = 1138*				
0.99	0.99	0.03	0.07	0.05 – 0.10
Family support[12], *1 factor, 5 items, 1 additional item covariance, n = 1122*				
1.00	0.99	0.02	0.08	0.05 – 0.10
Family cohesion[12], *1 factor, 7 items, 1 additional item covariance, n = 1129*				
0.98	0.97	0.04	0.08	0.07 – 0.10
Positive self-esteem[5], *1 factor, 5 items, 1 additional item covariance, n = 1148*				
1.00	0.99	0.01	0.08	0.06 – 0.11
Negative self-esteem[5], *1 factor, 5 items, 0 additional item covariances, n = 1151*				
1.00	1.00	0.01	0.04	0.02 – 0.07
Rumination: Brooding[7,10], *1 factors, 7 items, 1 additional item covariance, n = 1139*				
0.99	0.98	0.03	0.06	0.05 – 0.08
Rumination: Reflection[7], *1 factor, 5 items, 1 additional item covariance, n = 1148*				
1.00	1.00	0.01	0.02	0.00 – 0.06
Distress tolerance[13], *1 factor, 5 items, 1 additional item covariance, n = 1149*				
0.98	0.96	0.04	0.14	0.12 – 0.17
Aggression[14], *1 factor, 4 items, 0 additional item covariances, n = 1156*				
1.00	0.99	0.03	0.03	0.00 – 0.07

Note. WLSMV = weighted least squares estimator with mean- and variance corrected test statistics and robust standard errors. CFI = Comparative fit index, TLI = Tucker-Lewis index, SRMR = Standardized root mean square residual, RMSEA = Root mean square error of approximation, CI = Confidence interval.

Box-and-whisker plots with individual data points for the RFs (except expressive suppression) and the general distress variable can be found in Figure 1. Location and dispersion values for the RFs and the general distress variable can be found in Table 2. Due to the lack of variability we dichotomized aggression and expressive suppression RFs. Due to

deviations from normality for some of the remaining eight RFs, we transformed these eight factor scores and the general distress variable using the nonparanormal transformation.[15]

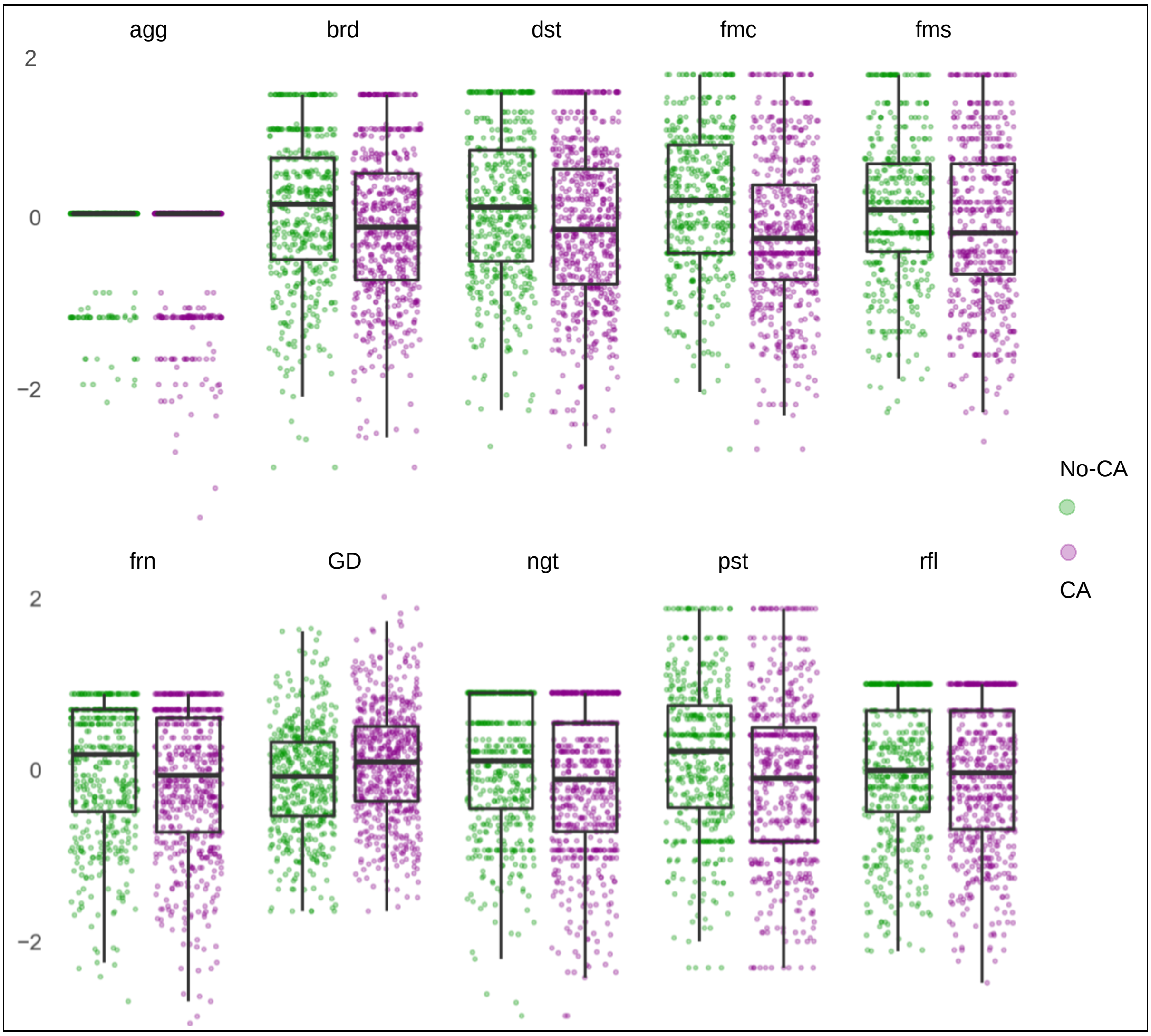

Figure 1. Box-and-whisker plots with individual data points for the untransformed RFs (except expressive suppression) and the general distress variable, separately for CA (*n* = 638) and no-CA (*n* = 501) groups. As expressive suppression contained three ordered categories (CA: 1 = 26, 2 = 183, 3 = 408; noCA: 1 = 12, 2 = 117, 3 = 366) we considered box-and-whisker plots with individual data points as inappropriate. No-CA group = green individual data points, CA group = magenta individual data points. Center line = median (50% quantile); lower box limit =25% quantile; upper box limit = 75% quantile; lower whisker = smallest observation greater than or equal to the lower box limit - 1.5 x Inter Quartile Range (IQR); upper whisker = largest observation less than or equal to upper box limit + 1.5 x IQR; outliers = data points beyond the end of the whiskers. **Legend:** Agg = aggression, brd = brooding, dst = distress tolerance, fmc = family cohesion, fms = family support, frn = friend support, ngt = negative self-esteem, GD = general distress, pst = positive self-esteem, rfl = reflective rumination.

Table 2

Means and Standard Deviations or Frequencies for Untransformed RF and the General Distress Variables of CA (n = 638) and No-CA (n = 501) Groups

	CA		***No-CA***	
**Variable*1/*2/*3**	***N***	**Mean(SD) / Median(IQR)**	***N***	**Mean(SD) / Median(IQR)**
Friendship support (high)	606	-0.13 (.82)	480	-0.01 (.76)
Family support (high)	585	-0.07 (.91)	481	0.08 (.85)
Family cohesion (high)	585	-0.16 (.90)	488	0.18 (.81)
Negative self-esteem (low)	610	-0.14 (.84)	488	0.05 (.77)
Positive self-esteem (high)	611	-0.12 (.95)	486	0.16 (.88)
Brooding (low)	604	-0.09 (.89)	486	0.07 (.87)
Reflective rumination (low)	608	-0.07 (.84)	487	-0.01 (.82)
Distress tolerance (high)	618	-0.12 (.91)	494	0.12 (.87)
Aggression (low)	613	-0.24 (.61)	491	-0.11 (.44)
Expressive suppression (low)	617	0.63 (1.86)	495	0.63 (1.86)
General distress	616	0.08 (.65)	490	-0.10 (.65)

Note. CA = childhood adversity, SD = standard deviation, IQR = inter quartile range. *1All RFs are scored in such a way that high values are protective (e.g. high levels of high friendship support or high levels of low negative self-esteem) and low values are harmful (e.g. low levels of high friendship support or low levels of low negative self-esteem). *2The continuous general distress variable is scored in such a way that the higher the value the higher the level of general distress. *3As expressive suppression contained three ordered categories we calculated the median and the inter quartile range, for all other variables the mean and the standard deviation were calculated.

Supplement III

Association Networks for CA and No-CA Groups

For the no-CA group, the association network (i.e. zero-order correlations; see Figure 2) showed that all RFs are positively correlated, except for the two relationships between expressive suppression and distress tolerance as well as expressive suppression and positive self-esteem. Interestingly, in the association network of the CA group (see Figure 2), expressive suppression was negatively associated with distress tolerance, reflective rumination, friendship support, and brooding.

**CA**	**No-CA**

2.a. Un-faded Association Networks

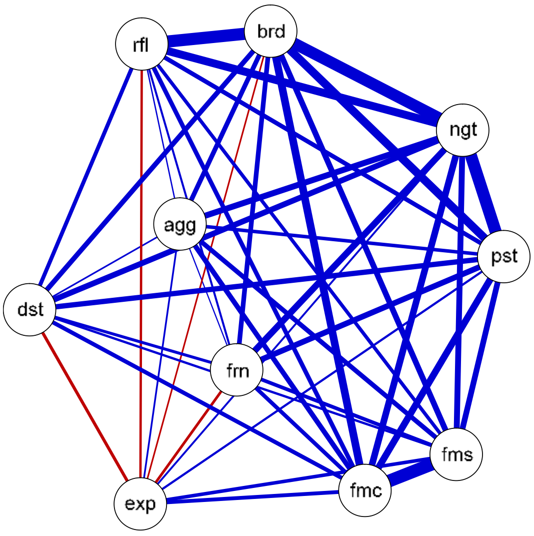

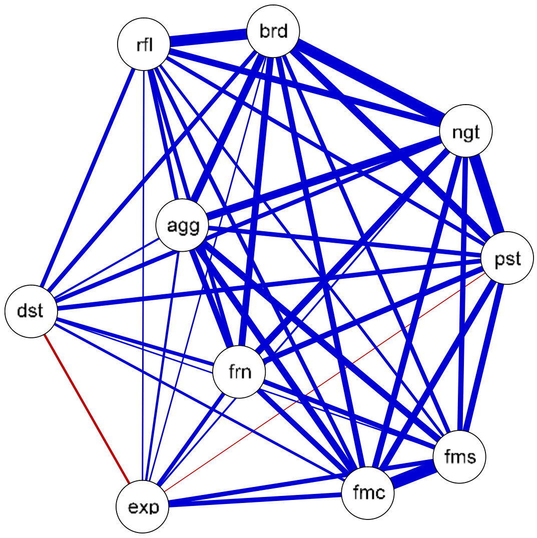

2.b. Faded Association Networks

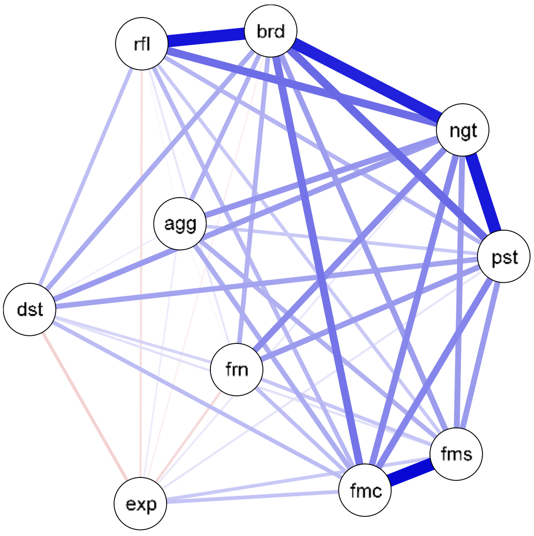

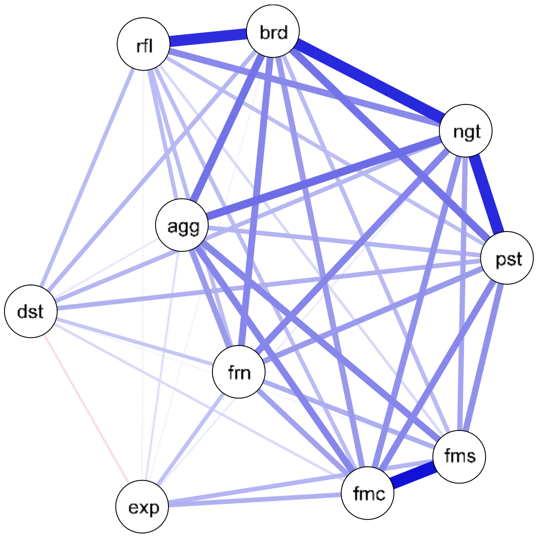

Figure 2. Association network for the CA (*n* = 638) and the no-CA (*n* = 501) group. Width of the lines = association strength. Positive interrelations = blue, negative interrelations = red. **Legend:** Frn = friend support, fms = family support, fmc = family cohesion, ngt = negative self-esteem, pst = positive self-esteem, rfl = reflective rumination, brd = brooding, dst = distress tolerance, agg = aggression, exp = expressive suppression.

Supplement IV

Adjacency Matrices of the Main Models

	friendship support	family support	family cohesion	positive SE	negative SE	brooding	reflection	distress tolerance	aggres-sion	exp. sup-press.
**1: Weights matrix for the lasso regularized RF network model of the CA group**										
friendship support	-	.023909	.028839	.080385	.148546	.000000	.000000	.000000	-.017070	-.071317
family support		-	.587941	.031853	.017381	.000000	.000000	.000000	.058560	.036042
family cohesion			-	.088599	.000000	.144786	.000000	.050993	.056685	.085883
positive SE				-	.455981	.009506	.000000	.078308	.000000	.013499
negative SE					-	.355060	.025007	.088398	.144010	.017677
brooding						-	.512462	.034055	.046442	.000000
reflection							-	.018345	-.058021	-.046307
distress tolerance								-	.000000	-.096202
aggres-sion									-	.000000
exp. sup-press.										-
**2: Weights matrix for the lasso regularized RF network model of the no-CA group**										
friendship support	-	.019055	.057729	.057601	.085972	.107758	.000000	.055556	.094934	.099539
family support		-	.548010	.078507	.000000	.000000	.000000	.000000	.117245	.070202
family cohesion			-	.088205	0.020039	.038283	.011123	.000000	.091018	.086695
positive SE				-	.424114	.015679	.000000	.094866	.000000	-.025461
negative SE					-	.356621	.000000	.024278	.147978	.000000
brooding						-	.450166	.033913	.134804	.000000
reflection							-	.070500	.000000	.000000
distress tolerance								-	.000000	-.060563
aggres-sion									-	.000000
exp. sup-press.										-
**3: Weights matrix for the lasso regularized RF network model of the CA group, including general distress**										
GD	-.195287	-.008303	-.089868	-.156796	-.374769	-.351656	-.046490	-.093833	-.228264	.062298
friendship support	-	.038692	.013288	.041158	.073642	.000000	-.106482	.000000	-.125764	-.106961
family support		-	.606414	.042512	.000000	.000000	.000000	-.041521	.066804	.041998
family cohesion			-	.058362	-.025743	.097499	.000000	.082392	.041399	.118914
positive SE				-	.375004	.000000	-.084934	.071999	-.082701	.039164
negative SE					-	.134735	.069420	.050035	.088657	.089301
brooding						-	.493546	.000000	.012984	-.014994
reflection							-	.018847	-.150017	-.065218
distress tolerance								-	-.044511	-.128313
aggres-sion									-	.000000
exp. sup-press.										-

4: Weights matrix for the lasso regularized RF network model of the no-CA group, including general distress

GD	-.055698	.000000	-.065887	-.096395	-.402710	-.366777	-.070095	-.020364	-.235897	.000000
friendship support	-	.019879	.054140	.044121	.059302	.077595	.000000	.048046	.075566	.090010
family support		-	.542330	.074108	.000000	.000000	.000000	.000000	.111047	.065862
family cohesion			-	.073279	.000000	.010217	.000000	.000000	.069631	.081735
positive SE				-	.331164	.000000	.000000	.086838	.000000	-.012585
negative SE					-	.139278	.000000	.012990	.026746	.000000
brooding						-	.370515	.024028	.027801	.000000
reflection							-	.060800	.000000	.000000
distress tolerance								-	.000000	-.051089
aggres-sion									-	.000000
exp. sup-press.										-

Note. CA = childhood adversity (yes: n = 638, no: n = 501); SE = self-esteem; Exp. suppress. = expressive suppression; GD = general distress.

Supplement V

Interconnectedness of RFs

In both the CA and the no-CA regularized partial correlation networks there were particularly strong positive relationships between high family cohesion and high family support (regularized partial correlation (reg-pcor) CA = .59, no-CA = .55), low brooding and low reflective rumination (reg-pcor CA = .51, no-CA = .45), low negative and high positive self-esteem (reg-pcor CA = .46, no-CA = .42), and between low brooding and low negative self-esteem (reg-pcor CA = .36, no-CA = .36). Interestingly, low expressive suppression was associated with *high* positive self-esteem and *low* friendship support in the CA network, which was reversed in the no-CA network (i.e. low expressive suppression with *low* positive self-esteem and *high* friendship support). Furthermore, in the CA network low aggression was associated with *low* friendship support, whereas the opposite pattern was revealed in the no-CA network (i.e. low aggression with *high* friendship support).

To examine the interrelatedness of the RFs, we calculated three coefficients. *Node strength* is the sum of the interrelation values (e.g. regularized partial correlations) of a given RF with all directly related RFs (i.e. the sum of the *absolute* values of the RF interrelations).[16,17] *Expected influence* is based on the formula of node strength, but takes negative relationships between RFs into account (i.e. the sum of the *relative* values of the RF interrelations).[17] *Node predictability* is defined as the amount of variance of each RF that is explained by the directly related RFs (i.e. *absolute* metric ranging from zero to 100 percent explained variance).[18] Node strength, expected influence and predictability had very similar RF importance rankings (Table 3). In sum, the self-esteem, brooding, and family RFs had the highest strength, expected influence and predictability values. Interestingly, low expressive suppression had a negative expected influence coefficient for the CA group (-0.06), but a positive coefficient for the no-CA group (0.17).

Table 3

Node Strength (S), Expected Influence (EI), Node Predictability (P), and the Belonging Coefficient Rank in Parenthesis, for Networks without the General Distress Variable

CA	negative SE	brooding	family cohesion	positive SE	family support	reflection	aggression	friendship support	expressive suppression	distress tolerance
					Strength					
	1.	*2.*	*3.*	*4.*	*5.*	*6.*	*7.*	*8.*	*9.*	*10.*
yes	1.25	1.10	1.04	0.76	0.76	0.66	0.38	0.37	0.37	0.37
no	1.06	1.14	0.94	0.78	0.83	0.53	0.59	0.58	0.34	0.34
	2.	*1.*	*3.*	*5.*	*4.*	*8.*	*6.*	*7.*	*9.*	*10.*
					Expected Influence					
	1.	*2.*	*3.*	*4.*	*5.*	*6.*	*7.*	*8.*	*10.*	*9.*
yes	1.25	1.10	1.04	0.76	0.76	0.45	0.23	0.19	-0.06	0.17
no	1.06	1.14	0.94	0.73	0.83	0.53	0.59	0.58	0.17	0.22
	2.	*1.*	*3.*	*5.*	*4.*	*8.*	*6.*	*7.*	*10.*	*9.*
					Predictability					
	2.	*1.*	*3.*	*6.*	*4.*	*5.*	*10.*	*7.*	*9.*	*8.*
yes	0.57	0.61	0.54	0.42	0.48	0.44	0.00	0.14	0.07	0.11
no	0.52	0.53	0.48	0.42	0.47	0.34	0.02	0.18	0.02	0.08
	2.	*1.*	*3.*	*5.*	*4.*	*6.*	*9.*	*7.*	*10.*	*8.*

Note. CA = Childhood adversity (yes: *n* = 638, no: *n* = 501). SE = Self-esteem.

Supplement VI

Robustness Analyses: Accuracy and Stability of the RF Network Models

To test the accuracy of the regularized partial correlation RF models we bootstrapped the RF interrelations (*N* boot = 2000) and to test the stability of the *node strength* and *expected influence* coefficients we applied a subset bootstrap (*N* boot = 2000). For CA and no-CA groups, family support and family cohesion had the highest interrelation, followed by reflective rumination and brooding, negative and positive self-esteem, as well as by negative self-esteem and brooding (Figure 3). Additional analyses showed that these four RF interrelations differed significantly from all other RF interrelations. The bootstrapped interrelation CIs had an acceptable width and we concluded that our models had a sufficient RF interrelation accuracy. With regard to the *node strength* and *expected influence* stability, we found for the CA network that up to 74.9 percent of the sample could be dropped to reveal (with a 95 percent likelihood) an association of minimal 0.7 between the subset and the original *node strength* (or *expected influence*) coefficients. This subset dropping percentage, of both *node strength* and *expected influence*, was 75 for the no-CA network. Therefore, we concluded that our models had a sufficient stability of the *node strength* and *expected influence* coefficients.

3.a. RF Interrelation Accuracy for the CA Group

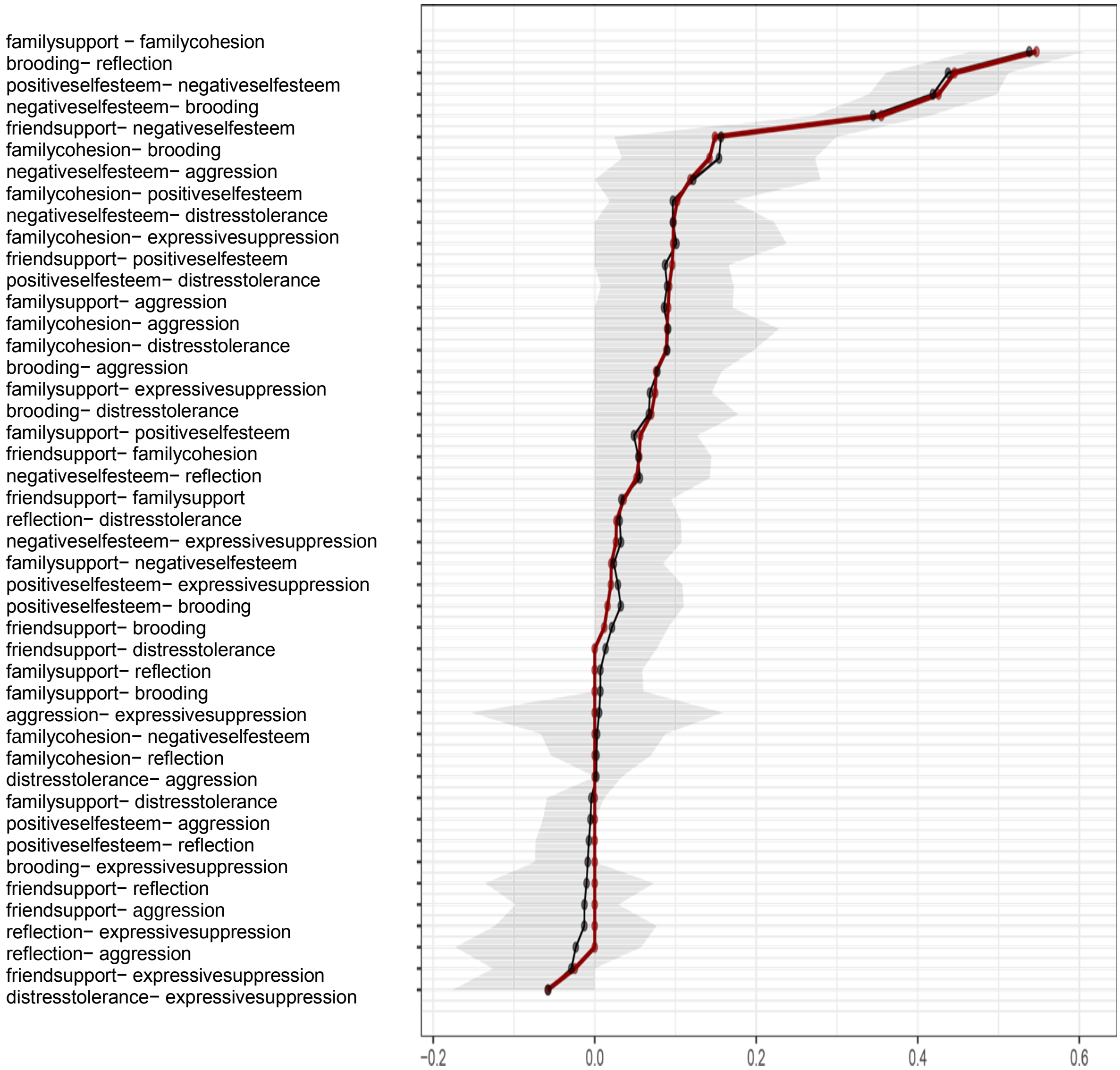

3.b. Node Strength and Expected Influence Stability for the CA Group

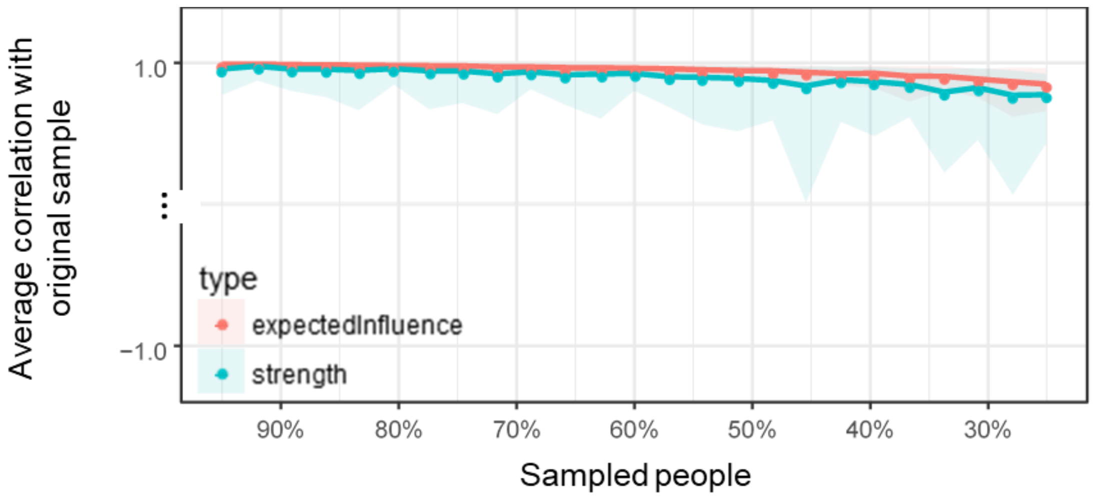

3.c. RF Interrelation Accuracy for the No-CA Group

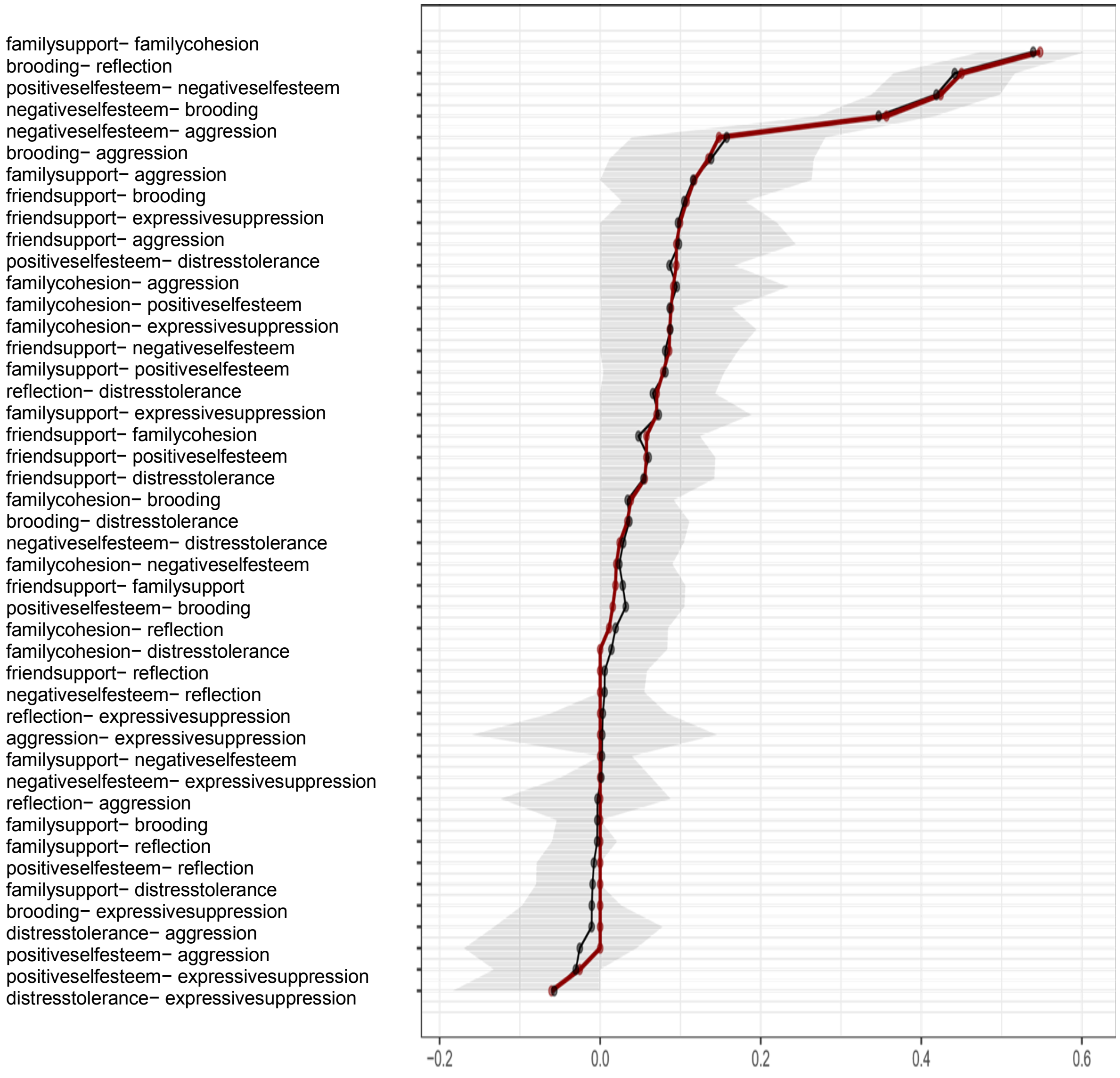

3.d. Node Strength and Expected Influence Stability for the No-CA Group

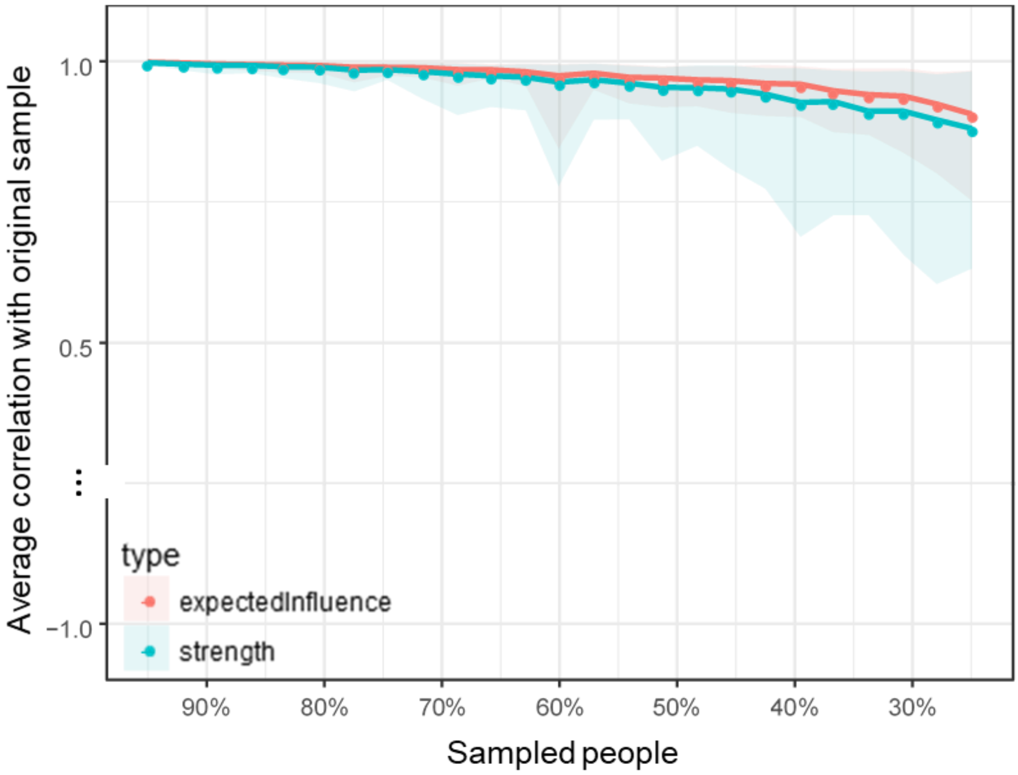

Figure 3. RF interrelation accuracy (3.a. and 3.c.) and node strength and expected influence stability (3.b. and 3.d.) plots for CA and no-CA groups. Panel '3.a.' and '3.b.' depict the CA (*n* = 638) and panel '3.c.' and '3.d.' the no-CA plots (*n* = 501). Panel '3.a.' and '3.c.' depict the sample RF interrelations (i.e. edge weights) which are represented by the red dots, the means of the bootstrapped RF interrelations (i.e. edge weights) which are represented by the black dots, and the belonging bootstrap confidence intervals (CIs) which indicate the RF interrelation accuracy. Panel '3.b.' and '3.d.' depict the average correlation of the node strength (and expected influence) coefficients between the original sample and the sample subsets.

Supplement VII

Sensitivity Analyses: Statistical Soundness of the RF Network Models

To allow for the largest possible sample size we based the network models on the full-information sample, using all possible pairwise correlations. This led to the result that different RF interrelation coefficients are based on different sample sizes. To substantiate the feasibility of this approach, we tested the extent to which the RF interrelations of the full-information (*N* CA = 638; *N* no-CA = 501) and the complete-information (*N* CA = 508; *N* no-CA = 443) samples are associated with each other. For both the CA and the no-CA group, the two regularized partial correlation networks were highly correlated (adjacency matrix correlation for CA: r = 0.99; for no-CA: r = 0.997). Similarly, the RF predictability networks (i.e. those models are not discussed in the text, but were established for the calculation of the predictability coefficients) had to be based on the complete-information subsets of the two samples (CA and no-CA). Therefore, we also scrutinized the relationship between the RF interrelations of the full-information regularized partial correlation networks and the predictability networks. Those RF interrelations were also highly correlated (adjacency matrix correlation for CA: r = 0.94; for no-CA: r = 0.97), indicating similarity between the results of the two methods.

Given that we pre-processed the RF variables through establishing factor scores and through applying transformations (i.e. nonparanormal method), we additionally performed sensitivity analyses to test the similarity of the reported regularized partial correlation networks with networks using (1) factor scores without transformation, (2) mean scores with transformation, and (3) mean scores without transformation. As all three additional models correlated highly with our reported models for the CA and the no-CA groups (which were based on factor scores with transformation), we concluded that our results are robust for the scrutinized sample (Table 4).

Table 4

Sensitivity Analysis for the Regularized Partial Correlation Network Models Being Based on Factor Scores and Making Use of the Nonparanormal Transformation

		**Reference model: factor scores, with transformation; comparison model:**		
**CA**	**correlation type**	**factor scores, no transformation**	**mean scores, with transformation**	**mean scores, no transformation**
yes	Pearson	.999	.99	.99
	Spearman	.99	.97	.97
no	Pearson	.999	.99	.99
	Spearman	.995	.95	.92

Note. CA = Childhood adversity (yes: *n* = 638, no: *n* = 501).

Supplement VIII

Main Models Depicted with Faded 'RF-RF' and 'RF-General Distress' Interrelations

CA	No-CA

4.a. RF Networks

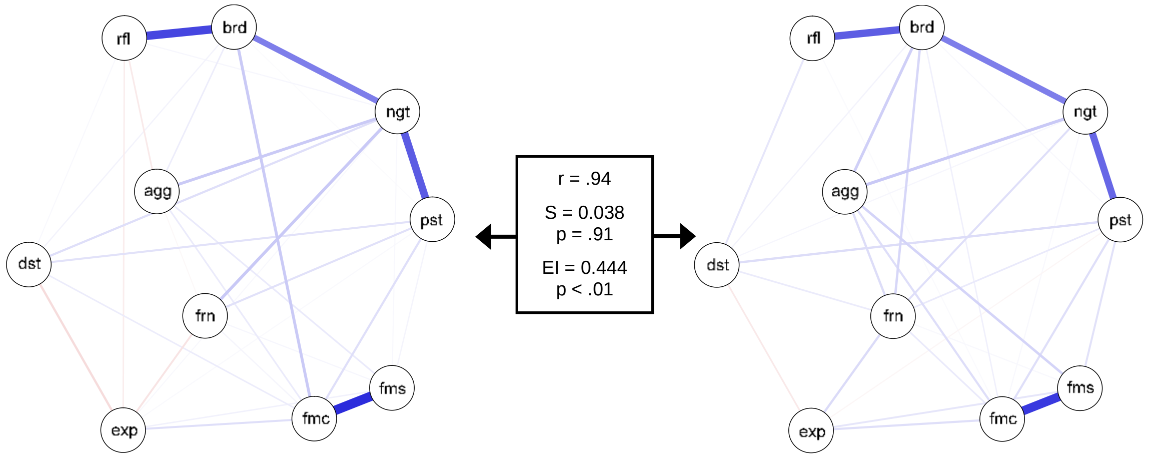

4.b. RF Networks with the General Distress Variable

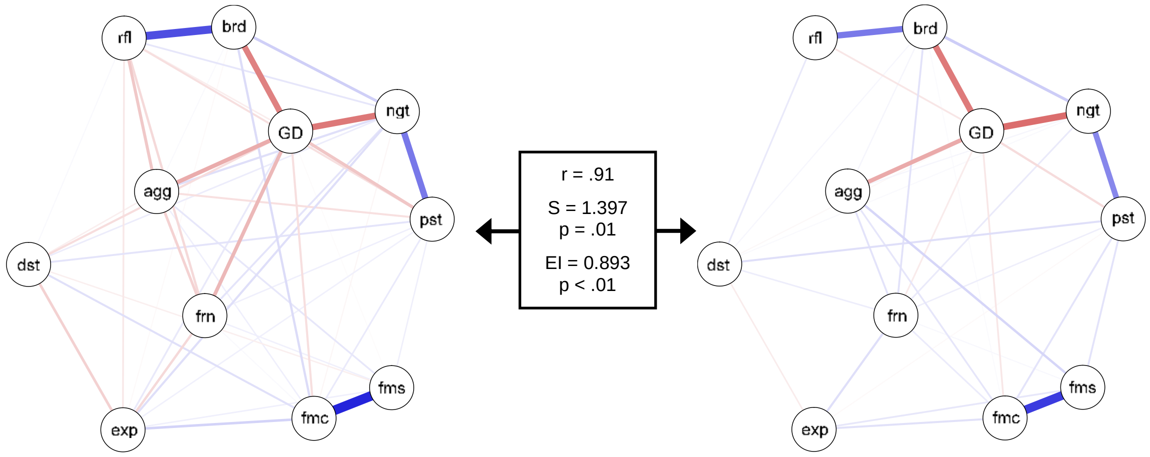

4.c. RF Networks Corrected for the General Distress Variable

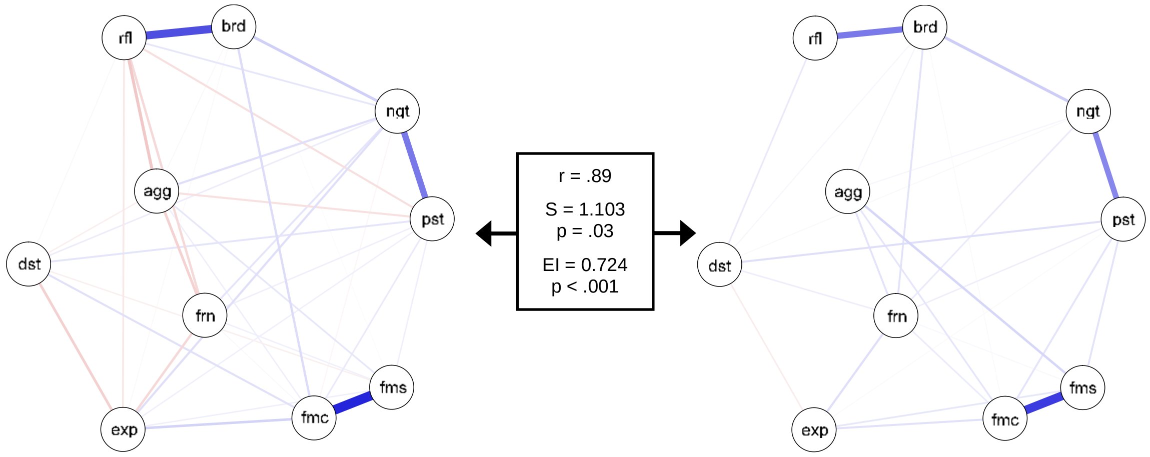

Figure 4. CA (*n* = 638) and no-CA (*n* = 501) resilience factor networks without (1.a.), with (1.b.), and corrected for (1.c.) the general distress variable. Width of the lines = association strength. Positive interrelations = blue, negative interrelations = red. **Legend:** Frn = friend support, fms = family support, fmc = family cohesion, ngt = negative self-esteem, pst = positive self-esteem, rfl = reflection, brd = brooding, dst = distress tolerance, agg = aggression, exp = expressive suppression, GD = general distress. The boxes depict the adjacency matrix correlation between the respective two networks (r), the difference in global network strength between the respective two networks (S), the difference in global network expected influence (EI) between the respective two networks (EI), and the p-value corresponding to the global network strength and global network EI comparisons (5000 comparison samples).

Supplement IX

Exploring the Influence of Expressive Suppression on the RF Networks of Each Group

For the no-CA group the expressive suppression RF was only in the association (i.e. zero-order correlations), but not in the regularized partial correlation network negatively associated with the general distress variable. For the CA group the expressive suppression RF was positively related with the general distress variable in both the association (i.e. zero-order correlation) and the regularized partial correlation network (see main text Table 2). As, in the CA group, expressive suppression had a positive zero-order correlation (i.e. relationship which is not corrected for the impact of the other RFs) with general distress (shown in bold in main text Table 2.b.), the unexpected relationship sign is not the result of correcting for the other RFs.

Further exploratory analyses revealed that expressive suppression (in isolation) neither moderated (interaction effect: b = 0.10, SE = 0.13, t = 0.75, p = 0.45), nor mediated (indirect effect = -0.001, SE = 0.005, z = -0.22, p = .82, CI[-.012, .009]) the relationship between CA and the general distress variable. Hence, in our sample, expressive suppression functioned (cross-sectionally) neither as a direct resilience factor nor as a direct risk factor for general distress after CA, when disregarding the impact of the other RFs.

Based on this finding and on the fact that expressive suppression was (in contrast to the other RFs) assessed with a single item, we re-estimated the regularized partial correlation RF networks for CA and no-CA groups this time without the expressive suppression variable (Figure 5). As in the models including expressive suppression, the relationship between aggression and friendship support was negative in the CA network, but positive in the no-CA network. Moreover, both the CA and the no-CA network revealed strong positive relationships between high family cohesion and high family support, low brooding and low reflective rumination, low negative and high positive self-esteem, as well as between low brooding and low negative self-esteem. Along those lines, the self-esteem, brooding, and family RFs had the highest strength and expected influence values. In sum, the RF networks without the expressive suppression variable resembled the corresponding networks including the variable.

The new regularized partial correlation networks of the CA and the no-CA group were highly correlated (correlation between the 36 regularized RF interrelations of each group: r = 0.95). Moreover, the network structure invariance test was not significant (M = .12, N permutations = 5000, p = 0.74), and the new CA and no-CA networks did neither differ with regard to the global network strength (S = 0.059, S_{CA} = 3.178, $S_{no\text{-}CA}$ = 3.237, N permutations = 5000, p = 0.86), the global network expected influence (EI; EI = 0.228, EI_{CA} = 3.009, $EI_{no\text{-}CA}$ = 3.237, N permutations = 5000, p = 0.08), nor with regard to single interrelation differences (36 tests, Holm-Bonferroni corrected: N permutations = 5000, corrected p > 0.05). As in the networks including expressive suppression, the degree of RF enhancement (i.e. 'global network EI') was higher in the no-CA than in the CA network, yet, in the new networks this difference did not reach significance.

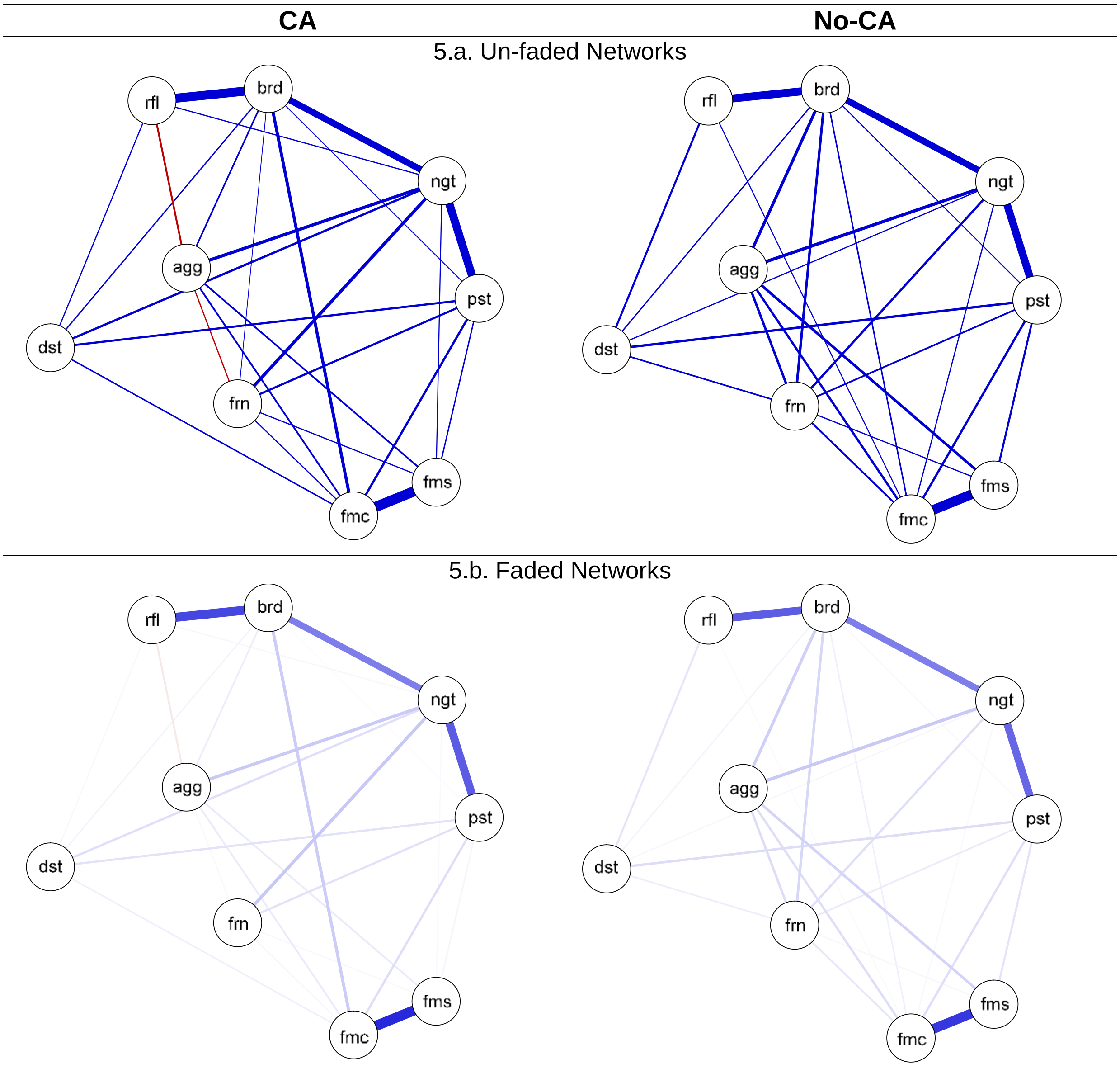

Figure 5. Regularized partial correlation network without the expressive suppression variable for the CA (*n* = 638) and the no-CA (*n* = 501) group. Width of the lines = association strength. Positive interrelations = blue, negative interrelations = red. **Legend:** Frn = friend support, fms = family support, fmc = family cohesion, ngt = negative self-esteem, pst = positive self-esteem, rfl = reflective rumination, brd = brooding, dst = distress tolerance, agg = aggression.

Supplement X

RF Interrelatedness Coefficients based on Networks Corrected for the General Distress Variable

Node strength and expected influence coefficients changed slightly in both groups, when taking general distress levels into account (correlation between the 10 RF coefficients of the networks without the distress variable and the networks corrected for the variance of the distress variable; *node strength*: CA r = .75, no-CA r = .79; *expected influence*: CA r = .93, no-CA r = .84). Importantly, the coefficient ranks changed notably after correcting for the general distress variable (see change in the coefficient rank order from Table 3 to Table 5).

Table 5

Node Strength (S), Expected Influence (EI), and the Belonging Coefficient Rank in Parenthesis, for Networks Corrected for the General Distress Variable

**CA**	**negative SE**	**brooding**	**family cohesion**	**positive SE**	**family support**	**reflection**	**aggression**	**friendship support**	**expressive suppression**	**distress tolerance**
					Strength					
	3.	6.	1.	5.	4.	2.	7.	9.	8.	10.
yes	0.91	0.75	1.04	0.80	0.84	0.99	0.61	0.51	0.61	0.44
no	0.57	0.65	0.83	0.62	0.81	0.43	0.31	0.47	0.30	0.28
	5.	*3.*	*1.*	*4.*	*2.*	*7.*	*8.*	*6.*	*9.*	*10.*
					Expected Influence					
	2.	*4.*	*1.*	*5.*	*3.*	*6.*	*10.*	*9.*	*8.*	*7.*
yes	0.86	0.72	0.99	0.46	0.76	0.18	-0.19	-0.17	-0.03	0.01
no	0.57	0.65	0.83	0.60	0.81	0.43	0.31	0.47	0.17	0.18
	5.	*3.*	*1.*	*4.*	*2.*	*7.*	*8.*	*6.*	*10.*	*9.*

Note. CA = Childhood adversity (yes: *n* = 638, no: *n* = 501). SE = Self-esteem.

Supplement XI

Network Pathways between the RFs and General Distress

We investigated the Shortest Paths Lengths (‘shortest pathways’) between the RFs and the general distress variable, for both the CA and the no-CA networks. The shortest pathway between two variables indicates the direct or indirect connection between those two variables along the strongest connection(s), or in other words the ‘quickest’ way between the two variables. Hence, shortest pathways designate whether the RFs have a direct connection with the general distress variable, or an indirect connection via other RFs. We found that the pathways between the RFs and the general distress variable differed for as many as 50 percent of the RFs. The five shortest pathways that differed between the CA and the no-CA group can be seen in the main text Figure 2 and the five shortest pathways that were equivalent can be found in Figure 6. In the CA group friendship support, family cohesion, and distress tolerance had direct shortest pathways with the general distress variable, whereas in the no-CA group these shortest pathways went via intermediate RFs (see Figure 2 in main text). Moreover, the shortest pathway for family support and the general distress variable went in the CA group via family cohesion and in the no-CA group via aggression. Similarly, the shortest pathway for expressive suppression and the general distress variable went in the CA group via negative self-esteem, and in the no-CA group via friendship support and brooding. In both CA and no-CA networks negative self-esteem, brooding and aggression had a direct shortest pathway with the general distress variable (see Figure 6). Moreover, in both CA and no-CA networks the shortest pathway between reflective rumination and the general distress variable went via brooding, and the shortest pathway between positive self-esteem and the general distress variable went via negative self-esteem. In sum, in the CA group six RFs had a direct shortest pathway with the general distress variable, whereas in the no-CA group only three RFs had a direct shortest pathway.

6.a. CA	6.b. No-CA

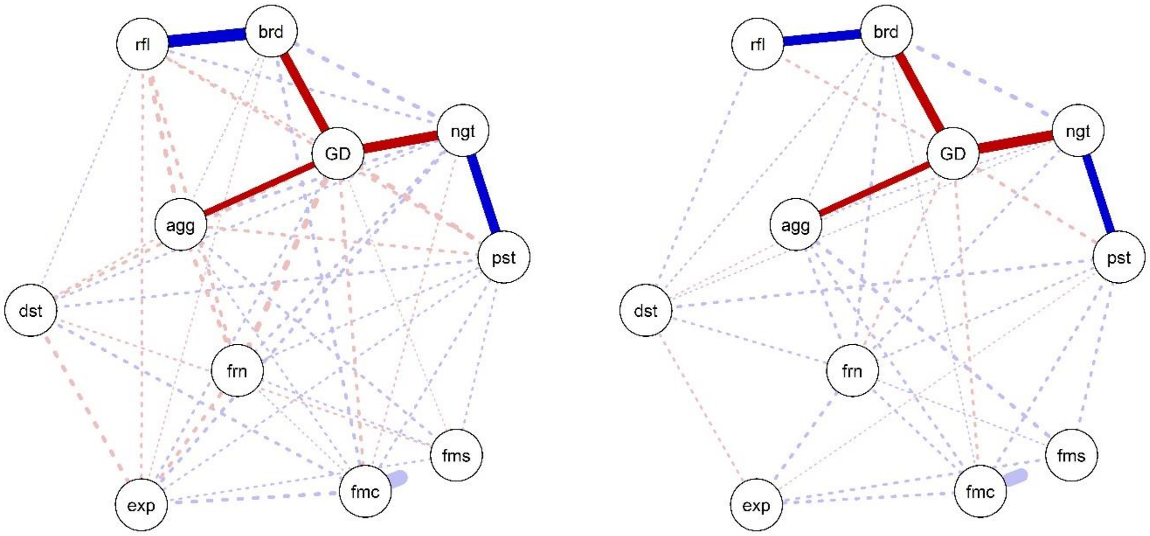

Figure 6. Shortest Path Length pathways (‘shortest pathways’) between the RFs and the general distress variable, that are equivalent between the CA (*n* = 638) and the no-CA (*n* = 501) group. Panel ‘6.a.’ depicts the CA network and panel ‘6.b.’ the no-CA network. Non-transparent, continuous lines = shortest pathway of interest. Transparent, dotted lines = all remaining regularized partial correlation connections. Positive interrelations = blue, negative interrelations = red. **Legend:** Frn = friend support, fms = family support, fmc = family cohesion, ngt = negative self-esteem, pst = positive self-esteem, rfl = reflection, brd = brooding, dst = distress tolerance, agg = aggression, exp = expressive suppression, GD = general distress.

Supplement XII

Exploring the Complex Interplay between CA, RFs and General Distress

Interestingly, in the CA network seven RF-RF interrelations turned from absent to negative and three from positive to absent, upon controlling for the general distress variable. In the no-CA network three RF-RF interrelations turned from positive to absent. In other words, in the CA network about 27 percent of the RFs are negatively interrelated and about 53 percent are positively related, upon controlling for general distress. In contrast, in the no-CA network only four percent of the RF interrelations are negative and about 56 percent are positively interrelated, upon controlling for general distress. Thus, while in the CA network many negative related RFs may hamper each other, in the no-CA network hardly any RFs seem to hamper each other. This finding was additionally supported by the degree of RF enhancement coefficient (i.e. 'general network EI', after controlling for general distress), which was significantly higher in the no-CA ($EI_{\text{no-CA}}$ = 2.514) than in the CA group (EI_{CA} = 1.790; EI = 0.724, permutations = 5000, p < .001). One speculative implication may be that RFs that hamper each other may alter 'RF-mental distress' relations unfavourably, resulting in an increased risk for subsequent mental health problems. However, it is important to discuss potential other, statistical explanations. We decided to control the RF-RF interrelations for general distress (see Figure 7 panel a), to correct for potentially spurious interrelations between RFs that better can be accounted for by general distress.[19] However, when conditioning on general distress, the variable may contrary to our intention not have behaved as a confounder (as in Figure 7 panel a), reducing spurious interrelations between the RFs, but may have behaved as a collider (see Figure 7 panel b) and may have induced spurious relationships between RFs.[19] This may explain why in the CA network, seven RF interrelations that were previously absent, i.e. non-existent, became negative upon the correction for general distress. However, based on our cross-sectional data, which reveals undirected interrelations between variables (i.e. the directionality of the effect could go either way: RFs predict general distress, or vice versa), and not directed relations as in in Figure 7, we cannot with certainty draw conclusions about whether general distress behaved as expected as a confounder, or contrary to our intention as a collider.

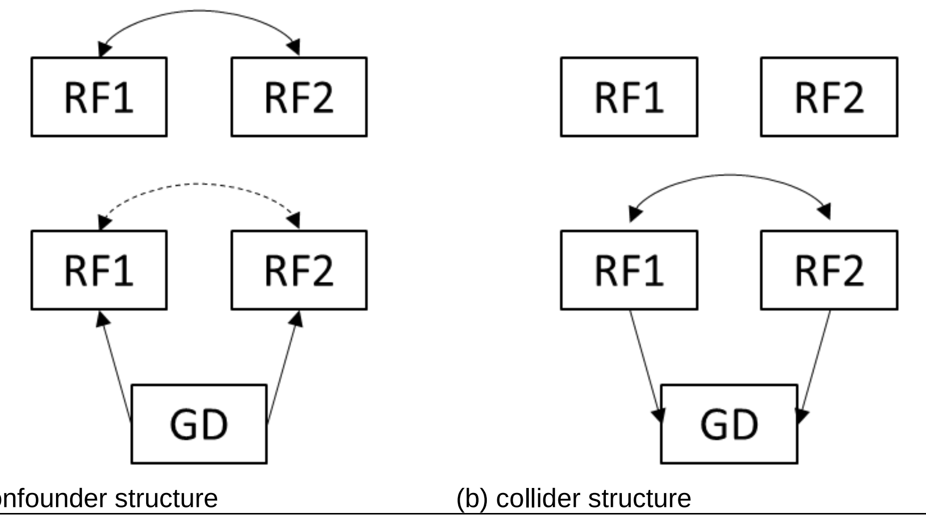

(a) confounder structure (b) collider structure

Figure 7. Conventional confounder and collider structures for potential effects of the general distress (GD) variable in relation to two resilience factors (RFs), presented within directed acyclic graph (DAG) networks. The Figures are modelled along discussed example of Elwert and Winship[19]; Annual Review of Sociology; can be retrieved from https://doi.org/10.1146/annurev-soc-071913-043455.

A priori, we expected that RFs would be more strongly related to general distress in the CA compared to the no-CA group. However, our results did not clearly show the expected pattern (see Table 2 in the manuscript). The zero-order correlations revealed that in the CA compared to the no-CA group, six RFs had slightly stronger, one RF an equally strong and

three RFs a slightly less strong interrelation with general distress. The regularized partial correlations revealed that in the CA compared to the no-CA group, five RFs had slightly stronger and five RFs a slightly less strong interrelation with general distress. Moreover, the interrelation strengths of the ‘RF-general distress’ interrelations also seemed to be rather comparable in the CA and the no-CA group (Pearson R = .92; Spearman R = .88). Therefore, we would have expected that correcting for general distress should have similar effects in both the CA and the no-CA network. Accordingly, we believe that conditioning on a collider is unlikely to be the main explanation for why conditioning on general distress seems overall to have different effects in the CA and the no-CA network.

Interestingly, even though single RFs were, in terms of interrelation strength, comparably related to distress in the two groups, we also showed that all except for one RF had significantly lower levels in the CA than in the no-CA group and that general distress was significantly higher in the CA compared to the no-CA group (see Table 1 in the manuscript). Thus in sum we found that (a) RFs are higher in the no-CA group, (b) distress is higher in the CA group, (c) ‘RF-general distress’ interrelations seem to be similarly strong in the two groups, but (d) correcting for distress seems to have differing effects in the two groups. More specifically, as ‘RF-general distress’ interrelations seem to be similarly strong in the two groups, it is surprising that the ‘RF-RF’ interrelations of the two groups, which also appear to be similar, seem to be differentially impacted by the correction of general distress. We speculate that the group differences may be the result of more complex interrelations between CA, RFs and general distress, such as underlying interaction (moderation) or indirect (mediation) effects. In our pre-registered systematic review,[20] we defined RFs as factors that mediate and/or moderate the relationship between CA and mental distress (i.e. different types or general measure of psychopathology/distress). Thus, as we feel that we cannot disentangle with certainty whether our general distress variable in our undirected models behaved as expected as a confounder or in contrast to our expectation as a collider, but as we can investigate other statistical explanations that may help explain and understand group differences, we decided to further explore whether the RFs (as expected) moderate and/or mediate the relationship between CA and general distress, cross-sectionally.

We believe that moderation effects seem less plausible for most RFs. For a moderation effect, the relationship between the RF and general distress would have to be significantly different for the CA and the no-CA groups, resulting in an interaction between the ‘RF-general distress’ slopes of the two groups (see Figure 8b). However, as the interrelations between the RFs and general distress seemed to be similarly strong in the CA and the no-CA group, and as the group slopes often in- or decreased in similar manners, significant interaction effects were unlikely. This conjecture was supported by our data. For example, Figure 8a depicts the ‘RF-general distress’ relationships between ruminative brooding and general distress first for the CA group, then for the no-CA group, and in the last panel for both the CA and the no-CA group. As can be seen, even if the CA group had overall higher levels of general distress at the same level of the RF, the pattern of relationship directionality (i.e. the slope) was similar for both groups. The only RF that revealed a significant interaction pattern was a low aggression potential (see Table 6a). Yet, this finding needs to be considered with caution, as both the CA and the RF variable were dichotomous, which is suboptimal for testing interactions. Moreover, the aggression interaction seemed to behave in the opposite direction than expected. A low aggression potential reduced general distress more in the no-CA than in the CA group (note bidirectionality). Thus, overall we conclude that moderation effects cannot explain the complex relationship between CA, RFs and general distress in our data.

Importantly, the revealed ‘RF-general distress’ interrelation pattern may well indicate mediation. More specifically, CA may negatively predict the RFs and the RFs in turn may negatively predict general distress. This would mean (a) that a history of CA goes together with a higher level of general distress, (b) that a history of CA leads on average to a lower level of RFs and (c) that higher levels of the RFs in turn lead to lower levels of general distress. All three necessary prerequisites of mediation were met by our data. Moreover, for mediation to hold, the relationship between an RF and general distress can have a similar directionality

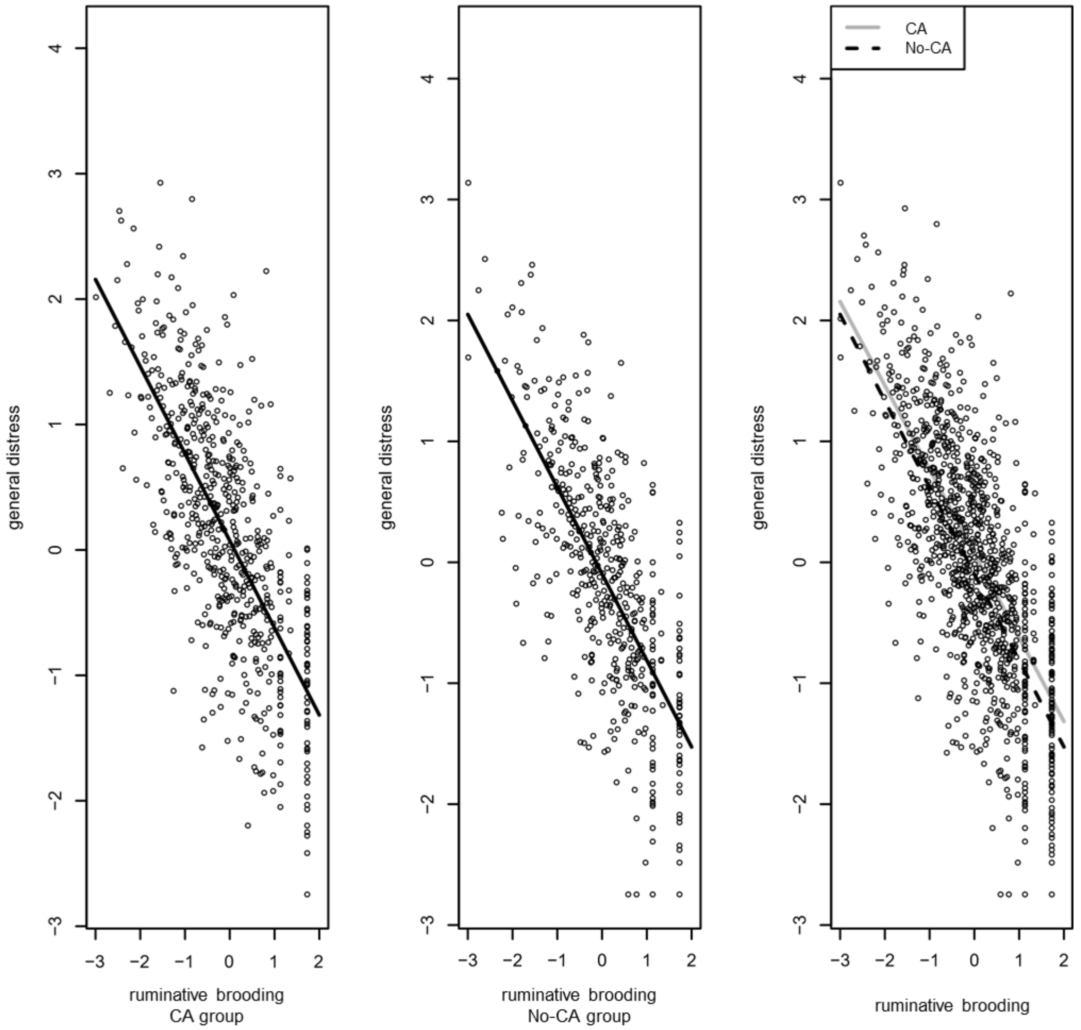

(a) Moderation: no significant interaction effect

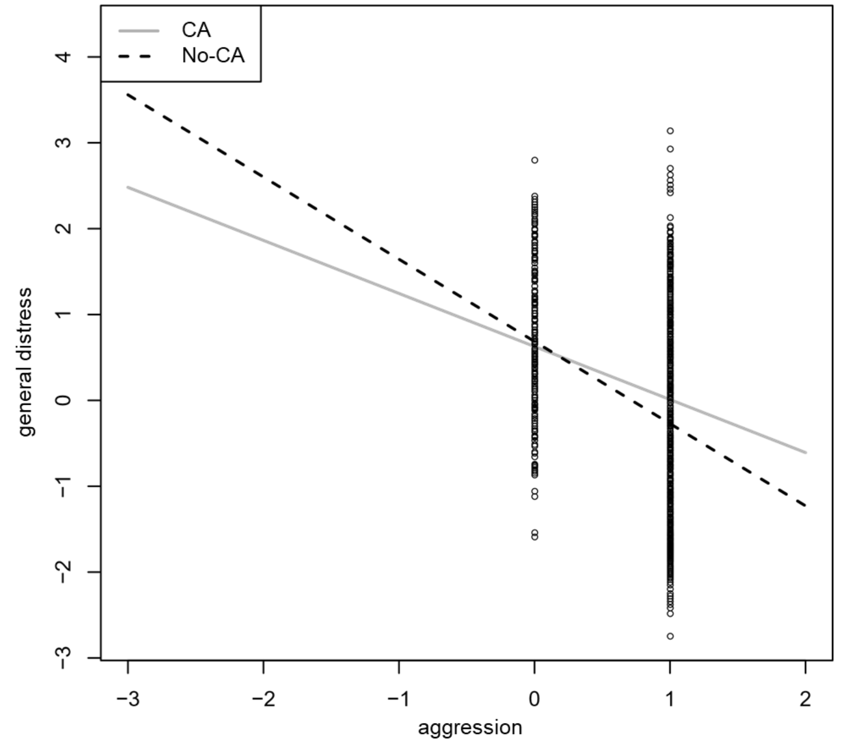

(b) Moderation: significant interaction effect

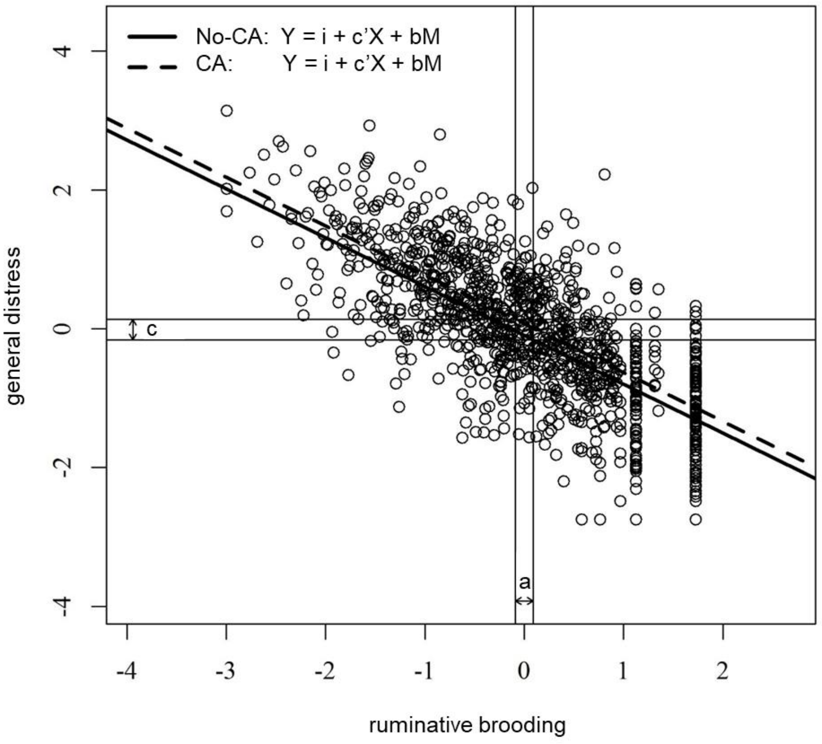

(c) Mediation: significant indirect effect

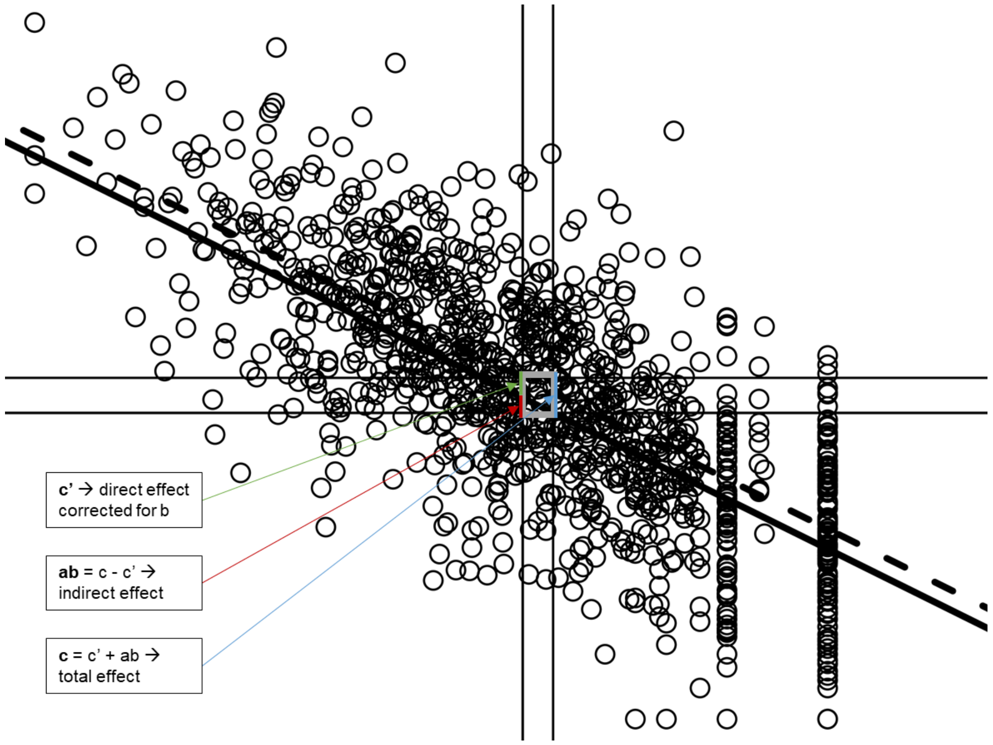

(d) Mediation: stipulation of the direct, indirect and total effects

Figure 8. Moderation and mediation example for RFs as moderator and mediator for the relationship between CA and general distress. Panel (a) depicts the RF low brooding, which has no significant interaction effect. Panel (b) depicts the RF low aggression potential, which has a significant interaction effect. Panel (c) depicts the RF low brooding, which has a significant indirect effect. Panel (d) stipulation of the direct, indirect and total effect of the mediation analysis for low brooding. The mediation figures are modelled using an adapted script from Fritz and MacKinnon[21]; Behavior Research Methods: https://doi.org/10.3758/BRM.40.1.55; the original scripts can be found in the belonging supplement material[22] at: https://link.springer.com/article/10.3758/BRM.40.1.55#SupplementaryMaterial.

pattern (i.e. slope) in the two groups, as long as either of the two groups has higher levels of the RF at the same level of general distress (see Figure 8c and 8d). This conjecture was clearly supported in our data. All RFs except for reflection and expressive suppression significantly mediated the relationship between CA and general distress (see Table 6b). Yet, to verify this conjecture, longitudinal approaches are necessary, as CA should be assessed no later than the RFs and the RFs should be assessed prior to general distress. However, the cross-sectional mediation effects may to some degree explain why the correction for distress levels had differing effects on the RFs in the CA compared to the no-CA group. Moreover, we believe that our conclusion that "CA seems to influence how resilience factors relate to each other and to current distress, potentially leading to a dysfunctional resilience factor system", was also supported by the post-hoc mediation findings, as those facilitate the idea of unfavourable 'RF-general distress' relations in the CA compared to the no-CA group, which may increase the risk for subsequent mental health problems.

Table 6

	friend support	family support	family cohesion	positive SE	negative SE	brooding	reflection	distress tolerance	aggres-sion	exp. sup-press.
**6a: RF as cross-sectional moderator for the relationship between CA and general distress**										
interaction effect est.	.02	-.04	-.02	-.01	.04	.02	.01	-.09	.34	.10
Std. error	.06	.06	.06	.05	.04	.04	.05	.06	.17	.13
t value	.32	-.61	-.32	-.24	.93	.49	.23	-1.44	2.04	.75
p (>|t|)	.75	.54	.75	.81	.36	.62	.82	.15	**.04***	.45
Adjusted analysis R^2	.15	.11	.17	.32	.56	.51	.22	.09	.09	.02
**6b: RF as cross-sectional mediator for the relationship between CA and general distress**										
indirect effect est.	.05	.05	.15	.17	.17	.13	.03	.07	.06	-.001
Std. error	.02	.02	.03	.03	.04	.04	.03	.02	.02	.01
z value	2.25	2.75	5.75	5.00	3.85	2.97	1.23	3.93	3.61	-.22
p value	**.03***	**.01****	**<.001*****	**<.001*****	**<.001*****	**.003****	.22	**<.001*****	**<.001*****	.82
95% *CI*	.01-.09	.02-.09	.10-.21	.10-.23	.08-.26	.04-.21	-.02-.09	.03-.10	.03-.09	-.01-.01
general distress R^2	.15	.11	.17	.32	.56	.52	.22	.09	.09	.02

Note. SE = self-esteem; Exp. suppress. = expressive suppression; Est. = estimate; Std. error = Standard error; CI = confidence interval.

Supplement XIII

Reliability and/or Validity Information for the used Measures

Name Variable	(Sub-)Scale/ Interview Used	Validity/ Reliability
Childhood adversity	Cambridge Early Experiences Interview (CAMEEI)[23]	• CAMEEI[23]; inter-rater reliability kappa = 0.7 - 0.9
General distress	Mood and Feeling Questionnaire[24] (MFQ; 33 items) + Revised Children's Manifest Anxiety Scale[25] (RCMAS; 28 items)	• MFQ[26]; internal consistency alpha = .94<br>• RCMAS[25]; KR_{20} reliability = .85
Friendship support	Cambridge Friendships Questionnaire[11] (CFQ; 5 of 8 items used)	• CFQ[27]; 2 weeks test retest reliability kappa = .80
Family support	General Functioning subscale of the McMaster Family Assessment Device[12] (GF-FAD; 5 of 12 items used)	• GF-FAD[12]; Cronbach's alpha = 0.92
Family cohesion	General Functioning subscale of the McMaster Family Assessment Device[12] (GF-FAD; 7 of 12 items used)	• GF-FAD[12]; Cronbach's alpha = 0.92
Positive self-esteem	Rosenberg self-esteem scale[5] (5 of 10 items used)	• RSES[28]; Cronbach's alpha = 0.88
Negative self-esteem	Rosenberg self-esteem scale[5] (5 of 10 items used)	• RSES[28]; Cronbach's alpha = 0.88
Reflective rumination	Ruminative Response Scale[7] (RRS; 5 of 22 items used)	• RRS[7]; Cronbach's alpha = .90;<br>• RRS 5 item subscale[7]; Cronbach's alpha = .72
Ruminative brooding	Ruminative Response Scale[7] (RSS; 5 of 22 items used) + Short Leyton Obsessional Inventory[10] (LOI; 2 of 11 items used)	• RRS[7]; Cronbach's alpha = .90;<br>• RRS 5 item subscale[7]; Cronbach's alpha = .77<br>• LOI[10]; internal reliability alpha = .94
Distress tolerance	Emotionality subscale of the Emotionality Activity Sociability Temperament Survey[13] (EAS; 5 items)	• EAS emotionality subscale[13]; Cronbach's alpha = .84 - 85
Aggression	Behaviour Checklist[14] (BC; 4 of 11 items)	• BC[29]; Cronbach's alpha = .74
Expressive suppression	Callous-unemotional subscale of the Antisocial Process Screening Device[30] (CU-APSD; 1 of 6 items)	• CU-APSD[30]; Cronbach's alpha = .22 - .60, with a median of .46

Supplement XIV

Individual RF Interrelation Differences between the CA and the no-CA Networks

Significant (and marginally significant) differences between individual RF interrelations of the CA and the no-CA networks (i.e. compared to the same individual RF interrelation differences between permuted network model pairs), before and after Holm-Bonferroni correction (see Table 7).

Table 7

Name RF1	Name RF2	Difference estimate	p-value
**RF Networks**			
After correction			
Friendship support	Expressive suppression	0.17	<.01
Before correction			
Friendship support	Brooding	0.11	.03
Friendship support	Aggression	0.11	.04
Friendship support	Expressive suppression	0.17	<.001
Family cohesion	Brooding	0.11	<.01
Positive self-esteem	Expressive suppression	0.04	.07
**RF Networks with the General Distress Variable**			
After correction			
Friendship support	Reflection	0.11	.09
Friendship support	Expressive suppression	0.20	<.001
Before correction			
Friendship support	Brooding	0.08	<.01
Friendship support	Reflection	0.11	<.01
Friendship support	Aggression	0.20	<.01
Friendship support	Expressive suppression	0.20	<.001
Friendship support	General distress	0.14	<.01
Family cohesion	Negative self-esteem	0.03	.04
Family cohesion	Brooding	0.09	.05
Family cohesion	Distress tolerance	0.08	.07
Positive self-esteem	Reflection	0.09	.05
Positive self-esteem	Expressive suppression	0.05	.05
Negative self-esteem	Reflection	0.07	<.01
Negative self-esteem	Expressive suppression	0.09	.02
Brooding	Reflection	0.12	.02
Reflection	Aggression	0.15	.04
Reflection	Expressive suppression	0.07	.08
Expressive suppression	General distress	0.06	.09
**RF Networks Corrected for the General Distress Variable**			
After correction			
Friendship support	Reflection	0.11	.07
Friendship support	Expressive suppression	0.20	<.01
Before correction			
Friendship support	Brooding	0.08	<.01
Friendship support	Reflection	0.11	<.01
Friendship support	Aggression	0.20	<.01
Friendship support	Expressive suppression	0.20	<.001
Family cohesion	Negative self-esteem	0.03	.04
Family cohesion	Brooding	0.09	.05
Family cohesion	Distress tolerance	0.08	.07
Positive self-esteem	Reflection	0.09	.04
Positive self-esteem	Expressive suppression	0.05	.06
Negative self-esteem	Reflection	0.07	<.01
Negative self-esteem	Expressive suppression	0.09	.02
Brooding	Reflection	0.12	.01
Reflection	Aggression	0.15	.04
Reflection	Expressive suppression	0.07	.08

REFERENCES

1. Epskamp, S., Waldorp, L. J., Mõttus, R. & Borsboom, D. The Gaussian Graphical Model in Cross-sectional and Time-series Data. *Multivariate Behav. Res.* **0,** 1–28 (2018).
2. Epskamp, S., Rhemtulla, M. & Borsboom, D. Generalized Network Psychometrics: Combining Network and Latent Variable Models. *Psychometrika* **82,** 904–927 (2017).
3. Epskamp, S., Maris, G. K. J., Waldorp, L. J. & Borsboom, D. Network Psychometrics. in *The Wiley Handbook of Psychometric Testing: A Multidisciplinary Reference on Survey, Scale and Test Development* (eds. Irwing, P., Hughes, D. & Booth, T.) (Wiley, 2018).
4. Bringmann, L. F. & Eronen, M. I. Don't blame the model: Reconsidering the network approach to psychopathology. *Psychol. Rev.* **125,** 606–615 (2018).
5. Rosenberg, M. *Society and the Adolescent Self-Image*. (Princeton, NJ: Princeton University Press, 1965).
6. Tomas, J. M. & Oliver, A. Rosenberg's self-esteem scale: Two factors or method effects. *Struct. Equ. Model. A Multidiscip. J.* **6,** 84–98 (1999).
7. Treynor, W., Gonzalez, R. & Nolen-Hoeksema, S. Rumination reconsidered: A psychometric analysis. *Cognit. Ther. Res.* **27,** 247–259 (2003).
8. Beck, A. T., Ward, C. H., Mendelsohn, M., Mock, J. & Erbaugh, J. An Inventory for Measuring Depression. *Arch. Gen. Psychiatry* **4,** 561–571 (1961).
9. Beck, A. T., Steer, R. A. & Garbin, M. G. Psychometric properties of the Beck Depression Inventory: Twenty-five years of evaluation. *Clin. Psychol. Rev.* **8,** 77–100 (1988).
10. Bamber, D., Tamplin, A., Park, R. J., Kyte, Z. A. & Goodyer, I. M. Development of a Short Leyton Obsessional Inventory for Children and Adolescents. *J. Am. Acad. Child Adolesc. Psychiatry* **41,** 1246–1252 (2002).
11. Goodyer, I. M., Wright, C. & Altham, P. M. E. Recent friendships in anxious and depressed school age children. *Psychol. Med.* **19,** 165–174 (1989).
12. Epstein, N. B., Baldwin, L. M. & Bishop, D. S. The McMaster Family Assessment Device. *J. Marital Fam. Ther.* **9,** 171–180 (1983).
13. Bould, H., Joinson, C., Sterne, J. & Araya, R. The Emotionality Activity Sociability Temperament Survey: Factor analysis and temporal stability in a longitudinal cohort. *Pers. Individ. Dif.* **54,** 628–633 (2013).
14. Goodyer, I. M. *et al.* Improving mood with psychoanalytic and cognitive therapies (IMPACT): a pragmatic effectiveness superiority trial to investigate whether specialised psychological treatment reduces the risk for relapse in adolescents with moderate to severe unipolar dep... *Trials* **12,** 175 (2011).
15. Zhao, T., Liu, H., Roeder, K., Lafferty, J. & Wasserman, L. The huge Package for High-dimensional Undirected Graph Estimation in R. *J. Mach. Learn. Res.* **13,** 1059–1062 (2012).
16. Costantini, G. *et al.* State of the aRt personality research: A tutorial on network analysis of personality data in R. *J. Res. Pers.* **54,** 13–29 (2015).
17. McNally, R. J. Can network analysis transform psychopathology? *Behav. Res. Ther.* **86,** 95–104 (2016).
18. Haslbeck, J. M. B. & Waldorp, L. J. How well do network models predict observations? On the importance of predictability in network models. *Behav. Res. Methods* (2017). doi:10.3758/s13428-017-0910-x
19. Elwert, F. & Winship, C. Endogenous Selection Bias: The Problem of Conditioning on a Collider Variable. *Annu. Rev. Sociol.* **40,** 31–53 (2014).
20. Fritz, J., de Graaff, A. M., Caisley, H., van Harmelen, A.-L. & Wilkinson, P. O. A Systematic Review of Amenable Resilience Factors that Moderate and/or Mediate the Relationship between Childhood Adversity and Mental Health in Young People. *Front. Psychiatry* **9,** 230 (2018).

21. Fritz, M. S. & MacKinnon, D. P. A graphical representation of the mediated effect. *Behav. Res. Methods* **40,** 55–60 (2008).
22. Fritz, M. S. & MacKinnon, D. P. med_plots.R. Retrieved June 21, 2018 from Psychonomic Society Web Archive: www.psychonomic.org/archive (2008).
23. Dunn, V. J. *et al.* Profiles of family-focused adverse experiences through childhood and early adolescence: The ROOTS project a community investigation of adolescent mental health. *BMC Psychiatry* **11,** 109 (2011).
24. Messer, S. C., Angold, A. & Costello, E. J. Development of a Short Questionnaire for Use in Epidemiological Studies of Depression in Children and Adolescents: Factor Composition and Structure across Development. *Int. J. Methods Psychiatr. Res.* **5,** 251–262 (1995).
25. Reynolds, C. R. & Richmond, B. O. What I Think and Feel: A Revised Measure of Children's Manifest Anxiety. *J. Abnorm. Child Psychol.* **6,** 271–280 (1978).
26. Wood, A., Kroll, L., Moore, A. & Harrington, R. Properties of the Mood and Feelings Questionnaire in Adolescent Psychiatric Outpatients: A Research Note. *J. Child Psychol. Psychiatry* **36,** 327–334 (1995).
27. van Harmelen, A.-L. *et al.* Friendships and Family Support Reduce Subsequent Depressive Symptoms in At-Risk Adolescents. *PLoS One* **11,** e0153715 (2016).
28. Gray-Little, B., Williams, V. S. L. & Hancock, T. D. An Item Response Theory Analysis of the Rosenberg Self-Esteem Scale. *Personality and Social Psychology Bulletin* **23,** 443–451 (1997).
29. Kiddle, B. *et al.* Cohort profile: The NSPN 2400 Cohort: a developmental sample supporting the Wellcome Trust NeuroScience in Psychiatry Network. *Int. J. Epidemiol.* 1–9 (2017). doi:10.1093/ije/dyx117
30. Poythress, N. G. *et al.* Internal Consistency Reliability of the Self-Report Antisocial Process Screening Device. *Assessment* **13,** 107–113 (2006).
